# Prognostic implications of high- OXPHOS macrophages in gastric cancer: a single-cell transcriptomics and tumor microenvironment communication study

**DOI:** 10.3389/fonc.2025.1533990

**Published:** 2025-06-20

**Authors:** Ziyuan Lin, Yunyu Xu, Xiaohe Xu, Xinjun Lin, Lin Cheng, Qifeng Zhao

**Affiliations:** ^1^ Department of Cardiac Surgery, The Second Affiliated Hospital and Yuying Children’s Hospital, Wenzhou Medical University, Wenzhou, China; ^2^ Department of Cardiac Surgery, The First People’s Hospital, The First Affiliated Hospital of Huzhou Normal University, Huzhou, China; ^3^ Department of Pharmacy College, Henan University, Kaifeng, China; ^4^ Department of Basic Medicine College, Bengbu Medical University, Bengbu, China; ^5^ Department of Pediatrics, The Second Affiliated Hospital and Yuying Children’s Hospital, Wenzhou Medical University, Wenzhou, China

**Keywords:** gastric cancer, tumor microenvironment, single-cell sequencing, prognostic signature, tumor-associated macrophages, cell communication

## Abstract

**Background:**

Gastric cancer (GC) is characterized by heterogeneous tumor microenvironment (TME) with various cell types contributing to disease progression and patient outcomes. This study aims to dissect the single-cell transcriptomic landscape of GC, highlighting the role of tumor-associated macrophages (TAMs) and establishing a novel prognostic signature based on high oxidative phosphorylation (OXPHOS) macrophages.

**Methods:**

Single-cell sequencing data from paired GC and normal stomach tissues, obtained from the GEO database (GSE184198), were processed to reveal cellular heterogeneity and identify TAM subsets with high OXPHOS activity. Using the TCGA STAD dataset, survival analyses were conducted on 435 GC patients to establish a high-OXPHOS-macrophage-related prognostic signature.

**Results:**

We identified eight distinct cell types within the GC TME, indicating significant cellular heterogeneity. Macrophages, particularly TAMs, were found in greater numbers in tumor tissue, with the C3 macrophage subset exhibiting the highest OXPHOS score. A 19-gene high-OXPHOS-macrophage-related prognostic signature was constructed, stratifying patients into different risk categories with significant survival differences (P<0.05). NPC2, LY96, and TPP1 were identified as key macrophage-expressed markers, correlating with prognosis. Cell communication analysis revealed increased interaction in tumor tissues, especially involving NPC2, LY96, and TPP1 positive macrophages, which facilitated tumorigenesis and immune evasion.

**Conclusion:**

The high-OXPHOS-macrophage-related prognostic signature derived from scRNA-seq data provides valuable insights into GC patient stratification. NPC2, LY96, and TPP1, highly expressed in TAMs, were implicated in promoting tumor growth and immune escape, offering potential targets for novel therapeutic interventions.

## Introduction

Gastric cancer remains one of the leading causes of cancer-related mortality worldwide, with its pathogenesis attributed to a confluence of genetic, environmental, and lifestyle factors (1, 2). Despite advances in surgical and chemotherapeutic interventions, the prognosis for patients with advanced GC is poor, highlighting an urgent need to deepen our understanding of its molecular underpinnings and to develop new therapeutic strategies (3).

At the heart of GC progression lies the TME, which is composed of various cell types including immune cells, stromal cells, and the extracellular matrix (4). Within this ecosystem, macrophages emerge prominently, not just as passive bystanders but as active paracrine communicators that shape the TME’s dynamics (5). Tumor-associated macrophages (TAMs), in particular, have been recognized for their dualistic roles; they can either suppress or promote tumor development depending on their polarization states, known as pro-inflammatory M1 and immunosuppressive M2 phenotypes (6, 7). The M2-type TAMs have been correlated with poor prognosis in GC due to their association with tumor growth, angiogenesis, and suppression of antitumor immunity (8, 9).

Recent developments in single-cell RNA sequencing (scRNA-seq) have unveiled the complex cellular heterogeneity within the TME, providing insights at a resolution unattainable by previous bulk analyses. Such detailed depictions allow for the identification of specific cell subpopulations, including diverse macrophage subsets and their distinct genetic expression patterns that may underpin their varied functions in GC (10, 11). Cell communication analysis, as an effective tool for analyzing interactions between cells, can be combined with prognosis analysis of GC to explain the factors affecting GC prognosis from a more micro perspective.

In parallel, advances in genome-wide analyses have facilitated the construction of prognostic signatures. These signatures can predict disease outcomes more accurately by utilizing the expression levels of specific genes linked to survival and informing the heterogeneity of GC (12, 13). The connection between the genetic programs of TAMs, particularly those governing oxidative phosphorylation (OXPHOS), and their potential to serve as biomarkers for GC prognosis is under intense investigation. OXPHOS, a metabolic pathway typically linked with energy metabolism, has recently come to light for its role in determining macrophage function and tumor progression (14).

Moreover, the intricate communication networks established by chemokines, cytokines, and growth factors between TAMs and other cells in the TME are now recognized as vital contributors to GC’s pathobiology (15, 16). Understanding such intercellular communications is essential for unraveling how specific cell subsets, particularly macrophages, facilitate tumor progression and influence therapeutic responses (17).

To address these gaps in our knowledge, our study employed innovative techniques to map out the cellular architecture of the TME in GC and to identify potential prognostic markers correlating with patient outcomes. We devised a high-OXPHOS-macrophage-related prognostic signature based on scRNA-seq data, which we validated using the TCGA database. Our investigation into the intricate interplay between macrophages and the TME through the lens of NPC2, LY96, and TPP1 expression further cements the hypothesis that TAMs are central regulators in the precincts of gastric tumorigenesis.

## Methods

### Single-cell analysis

Single-cell sequencing data of paired gastric cancer tissue and normal stomach tissue were obtained from the GEO database (https://www.ncbi.nlm.nih.gov/geo; ID: GSE184198), including data from 1 gastric cancer sample and 1 normal stomach sample (18). To externally validate the results of the single-cell analysis, we obtained GSE268238 from the GEO database. The retrieved expression matrix was used to create a Seurat object, which was then matched with the acquired metadata. The data uploader had cleaned and filtered the data, so there is no need for further data cleaning (filtering thresholds: nCount_RNA > 1000 & nFeature_RNA < 5000 & percent.mt < 30 & nFeature_RNA > 600). The CellMarker database (http://xteam.xbio.top/CellMarker/) was used for manual annotation of cells (19). The harmony R package was used to remove batch effects between samples.

The subset function was used to extract a subset Seurat object from the Seurat object. The NormalizeData function was used to normalize the Seurat object. The FindVariableFeatures function was used to calculate highly variable genes. All cells were divided into different Seurat clusters at a resolution of 0.4. The RunUMAP function was applied to perform dimensionality reduction on the Seurat object. The genes related to oxidative phosphorylation were obtained from MsigDB (https://www.gsea-msigdb.org/gsea/msigdb), and the AddModuleScore function was utilized to evaluate the oxidative phosphorylation score of each cell. The R package ‘Seurat’ was used to perform the above analysis (20).

### Survival analysis

We conducted an analysis using the STAD data from the TCGA database (https://portal.gdc.cancer.gov/) and gastric cancer data (GSE62254) from GEO database (https://www.ncbi.nlm.nih.gov/geo/). After data cleaning and filtering, we obtained an expression matrix (TCGA) composed of 435 gastric cancer patients and their corresponding clinical data, and expression matrix (GEO) composed of 300 gastric cancer patients and their corresponding clinical data. Patients were divided into groups with high and low expression based on the median value of gene expression. The R package ‘survival’ was used to perform the survival analysis. The Kaplan-Meier (KM) analysis was utilized to determine survival differences, and a P-value of less than 0.05 was considered statistically significant.

### Construction of a prognosis-related signature

We used high-OXPHOS-macrophage-related genes screened by single-cell sequencing to construct a prognosis-related signature. Therefore, the final selection of genes used to construct the prognosis-related signature was associated with macrophages and high OXPHOS. The R package ‘survival’ was utilized for univariate Cox analysis and multivariate Cox analysis. KM analysis was employed to determine survival differences, with a P-value less than 0.05 considered statistically significant. After univariate and multivariate Cox analyses, the selected genes comprised the prognosis-related signature. The following formula was used to calculate the risk score:


$$risk\;score=\;\sum_{i}^{n}\exp\left(RNAi\right)*coef\left(RNAi\right)$$

The expression level of each RNA was denoted as exp (RNAi), and the multivariate Cox regression coefficient for each RNA was denoted as coef (RNAi). The R package ‘survivalROC’ was used to assess the accuracy of prognosis -related signature. The R package ‘ggplot2’ was used for data visualization.

### Cell communication analysis

Based on the expression level of NPC2, LY96 and TPP1, we classified all macrophages into gene-positive macrophages and gene-negative macrophages. A Seurat object was used to construct a CellChat object. After loading the human receptor-ligand pair database into the CellChat object, an intercellular interaction network was established based on the existing receptor-ligand pair information. The R package CellChat was used to carry out cell communication analysis, and the analysis was carried out according to the standard protocol document (21).

### Tissue microarray immunofluorescence

We acquired the tissue microarray from Origin Biotechnology Inc., which contained 80 gastric cancer tissues and 80 normal stomach tissues. We followed the standardized procedure provided by the company to perform immunofluorescence staining on the tissue microarrays. We used anti-NPC2 (Invitrogen, CAT#PA5-143858, 1:500), anti-LY96 (Invitrogen, CAT#MA5-15766, 1:200), anti-TPP1 (Invitrogen, CAT#PA5-22274, 1:200) and anti-CD68 (Invitrogen, CAT#14-0688-82, 1:500) as primary antibodies, with goat anti-mouse-488 (abcam, CAT#ab150113, 1:1000) and goat anti-rabbit-594 (abcam, CAT#ab150080, 1:1000) as secondary antibodies. After the antibody incubation was completed, we stained the cell nuclei with DAPI. Once all staining procedures were finished, we processed the tissue microarrays with a confocal microscope (ZEISS, LSM 980).

### OXPHOS inhibition in THP1 macrophages

THP1 cells were seeded into plate/dish (P3-P6, 1×10^4^ cells/ml) and cultured in RPMI 1640 medium (Servicebio, CAT #G4531) supplemented with 10% fetal bovine serum (FBS) (Servicebio, CAT #G8003), 0.05mM β-mercaptoethanol (MCE, CAT#HY-Y0326), and 1% penicillin/streptomycin (Servicebio, CAT#G4003). IACS-010759 (MCE, CAT#HY-112037) was used to inhibit OXPHOS in THP1 cells. The cells were treated with IACS-010759 (1.4 nM) for 48 hours and then fixed with paraformaldehyde. After fixation, the cells underwent immunofluorescence staining, following the same procedure as the aforementioned tissue immunofluorescence staining.

### Statistical analysis

The R software (version 4.2.2) was used for all analyses. Prism 9 was used for data analysis. Kolmogorov-Smirnov test was used to confirm normality of data. Student’s t-test was used to confirm statistical significance if data were normally distributed. Mann-Whitney U test was used to confirm statistical significance if data were not normally distributed. *P*< 0.05 was considered statistically significant.

## Results

### Gastric cancer single-cell atlas

**Figure 1:**
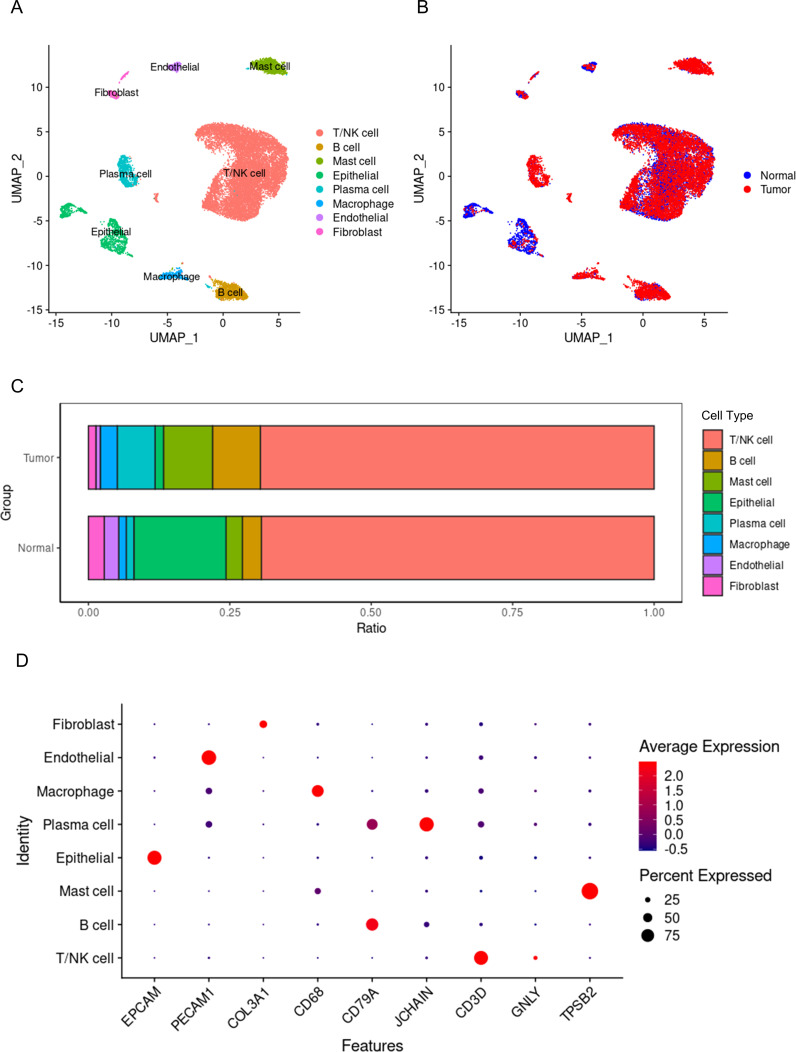
Single-cell panorama of gastric cancer. **(A)** the distribution of all cells, with points of different colors representing different cell types. **(B)** the distribution of all cells, with points of different colors representing different tissue origins. **(C)** the proportions of different cell types in tumors and normal tissues, with different colors representing different types of cells. **(D)** the expression of marker genes for different cell types, where blue points indicate low expression levels, red points indicate high expression levels, and the size of the point represents the percentage of cells expressing the gene out of all cells.

A total of 17,985 cells were classified into 8 cell types, including non-immune cells such as epithelial cells, endothelial cells, and fibroblasts, as well as immune cells such as T or NK cells, mast cells, B cells, plasma cells, and macrophages (**Figure 1A**). Epithelial cells originating from tumor tissue are considered to be malignant tumor cells, while those from normal tissue are normal functioning gastric epithelial cells. Similarly, macrophages from tumor tissue are considered to be tumor-associated macrophages, while those from normal tissue are deemed to have normal functions. After removing the batch effect between samples, the cells from tumor tissues and normal tissues exhibit a uniform distribution (**Figure 1B**). The proportions of the 8 different cell types also show significant differences between tumor and normal tissues (**Figure 1C**). The proportions of T or NK cells are nearly the same in both types of tissues, while the proportion of macrophages is significantly higher in tumor tissue compared to normal tissue. Therefore, it can be acknowledged to some extent that macrophages play a key role in the initiation and progression of tumors. **Figure 1D** displays the marker genes for each cell type.

### The single-cell transcriptomic landscape of macrophages in gastric cancer

**Figure 2:**
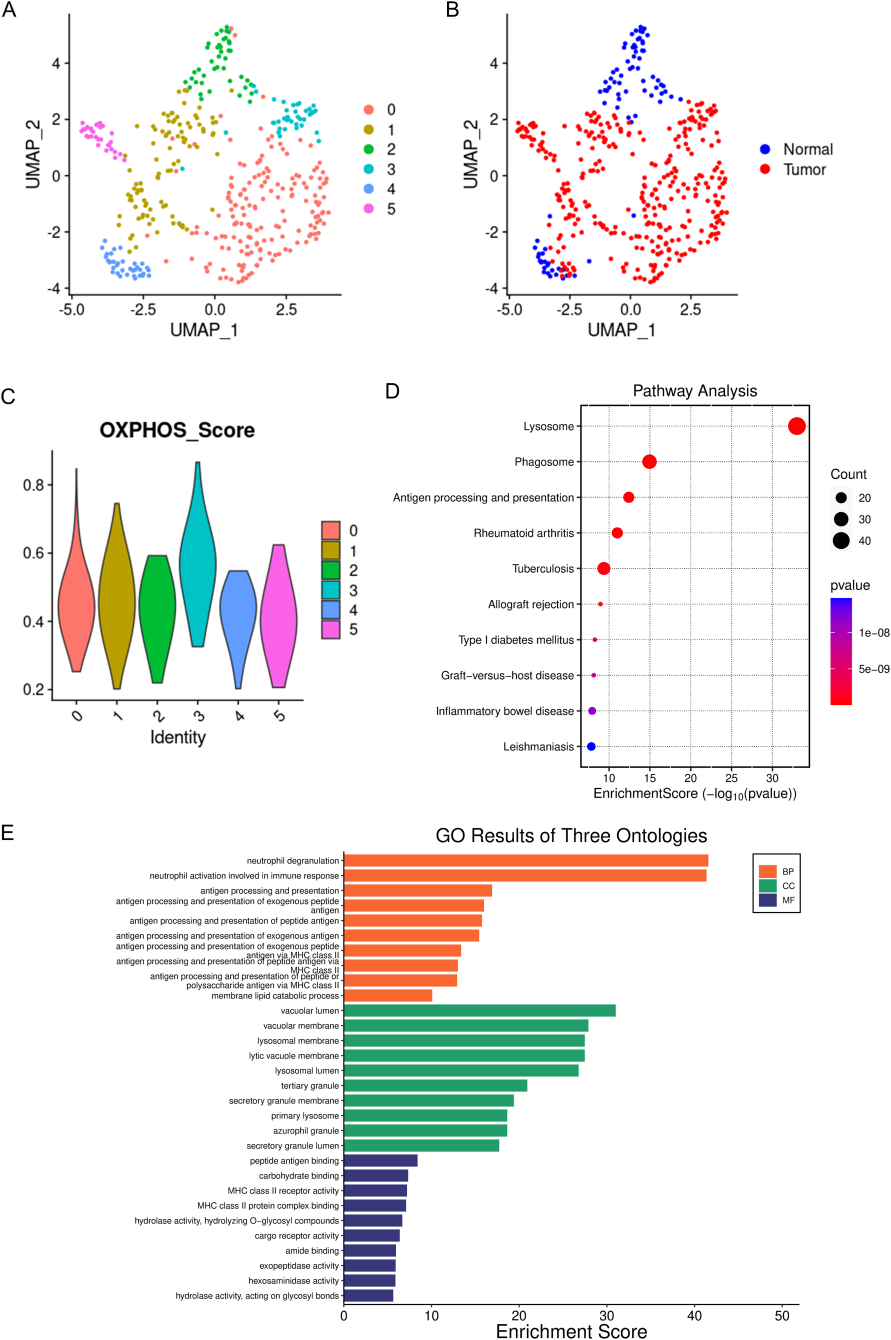
Single-cell panoramic view of macrophages. **(A)** dots of different colors represent cell clusters. **(B)** dots of different colors represent different tissue origins. **(C)** the oxidative phosphorylation scoring of different macrophage subgroups, with different colors representing different cell subgroups. **(D)** KEGG enrichment of the marker genes for C3 macrophages, where the size of the dots indicates the number of genes, and the color of the dots represents the significance of the enrichment. **(E)** GO enrichment of the marker genes for C3 macrophages.

A total of 956 macrophages were divided into 6 subgroups (**Figure 2A**). Among these, the proportion of TAMs originating from tumor tissues was significantly higher than that of macrophages derived from normal tissues (**Figure 2B**). Studies have indicated that TAMs are primarily M2-like macrophages, which have tumor-promoting functions (22–24). Furthermore, the metabolic characteristics of TAMs are marked by active oxidative phosphorylation that produces a large number of ligands that promote tumor invasion and growth (25, 26). Therefore, we calculated oxidative phosphorylation scores based on the expression levels of genes related to macrophage oxidative phosphorylation (**Supplementary Tables S1** and **S2**). We found that C3 macrophages had the highest oxidative phosphorylation scores (**Figure 2C**), suggesting that C3 macrophages may possess the capability to promote tumor invasion and growth. Subsequently, we extracted the signature genes of C3 macrophages and conducted Gene Ontology (GO) and Kyoto Encyclopedia of Genes and Genomes (KEGG) enrichment analyses. In the KEGG analysis, signature genes were significantly enriched in pathways related to the lysosome (**Figure 2D**). Research has confirmed that TAM lysosomes can release enzymes that dissolve the extracellular matrix, thereby enhancing the invasive ability of cancer cells (27, 28). This further substantiates the role of C3 macrophages in tumor promotion. In the GO analysis, the signature genes were also significantly enriched in lysosome-related functions (**Figure 2E**), further supporting the important role of C3 macrophages, consistent with the results above. C3 macrophages may affect the TME of GC.

### Construction of high-OXPHOS-macrophage related prognostic signature

**Figure 3:**
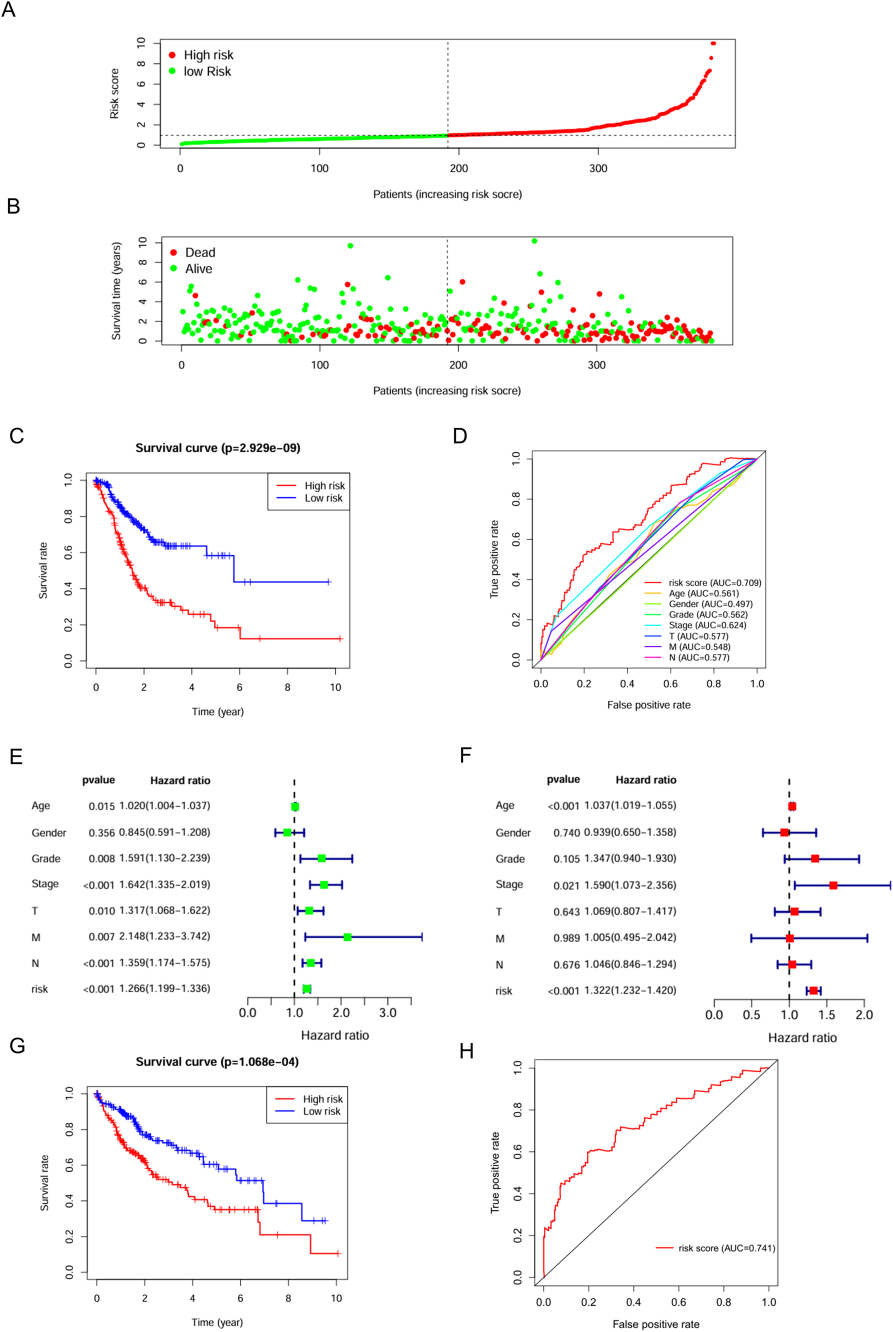
Assessment of the predictive effect of the prognostic signature. **(A)** risk curve displays the distribution characteristics of the risk scores. **(B)** scatter plot shows that the mortality rate of patients increases with higher risk. **(C)** survival curve for the risk scores calculated by the prognostic signature. **(D)** the ROC curve of the risk score and clinically relevant features. **(E)** forest plot shows the results of the univariate Cox analysis. **(F)** forest plot shows the results of the multivariate Cox analysis. **(G)** survival curve for the risk scores calculated by the prognostic signature (external validation). **(H)** the ROC curve of the risk score (external validation).

C3 macrophages had the highest OXPHOS score, thus marker genes of C3 macrophages would be analyzed as high-OXPHOS-macrophage related genes in the next step, with a total of 510 C3 macrophage marker genes. A total of 510 high-OXPHOS-macrophage related genes were screened out for further analysis. Univariate Cox regression analysis was used to filter the genes related to prognosis of gastric cancer, obtaining 50 prognosis-related genes (**Table 1**). Then, to identify more prognostically valuable genes, multivariate Cox regression analysis was conducted on the 50 prognostic-related genes, eventually yielding 19 prognostic genes (**Table 2**). Based on these 19 prognostic genes, a risk score was calculated for each patient. Patients were divided into high-risk and low-risk groups according to the median risk score. **Figure 3A** displays the distribution range of the risk scores. Patients with higher risk scores also had a higher risk of death (**Figure 3B**). Patients in the low-risk group had a higher survival rate compared to those in the high-risk group (**Figure 3C**). Moreover, compared to other clinical characteristics, the risk score achieved a higher AUC (**Figure 3D**), indicating that the high-OXPHOS-macrophage related prognostic signature has better predictive capability for prognosis. Similarly, the risk score demonstrated significant prognostic prediction abilities in both univariate and multivariate Cox regression analyses, further indicating that the risk score is an independent risk factor for gastric cancer (**Figures 3E, F**). Finally, we validated the risk model using an external gastric cancer dataset, further demonstrating the prognostic value of the risk model (**Figures 3G, H**). The high-OXPHOS-macrophage-related prognostic signature can effectively predict the prognosis of GC patients.

**Table 1:** Univariate Cox regression analysis of high-OXPHOS-macrophage related genes.

Gene	B	HR	*P*-value
A2M	0.00226	1.002263	0.001851
GPR34	0.06661	1.068878	2.30E-05
NRP1	0.068412	1.070807	1.87E-07
C1orf54	0.090987	1.095255	0.001381
DAB2	0.038002	1.038733	0.000455
NPC2	0.011371	1.011436	0.004166
TIMP2	0.006926	1.00695	0.001257
GNB4	0.068286	1.070671	0.001532
TCN2	0.027318	1.027695	0.015014
LRP12	0.257974	1.294305	0.008815
RNASE1	0.000827	1.000827	0.000431
VEGFB	0.010046	1.010097	0.011747
SPRED1	0.077489	1.08057	0.005504
ITM2B	0.009904	1.009953	0.004415
AKR1B1	0.026165	1.026511	0.000215
ENPP2	0.028261	1.028664	0.017977
ST14	-0.00433	0.99568	0.026575
LY96	0.027586	1.02797	7.10E-06
MFSD13A	-0.10097	0.903956	0.035135
TPP1	0.013563	1.013655	0.012772
MLEC	-0.00796	0.992074	0.024518
CLEC11A	0.016109	1.016239	0.03745
CALU	0.012077	1.01215	0.009663
MCRIP2	-0.04479	0.956195	0.048333
GSN	0.005629	1.005645	0.027144
PLBD2	0.023366	1.023641	0.019091
CPNE8	0.233742	1.263318	1.67E-05
OSBPL1A	0.095587	1.100305	0.001111
MFSD12	-0.04117	0.95967	0.007882
UBE2E2	0.04928	1.050514	0.045838
ELOVL1	-0.00988	0.990171	0.030783
PTGES2	-0.03959	0.961182	0.015752
SNX29	0.129335	1.138071	0.008139
BEX4	0.02051	1.020722	0.003553
CHCHD10	-0.00317	0.99683	0.017058
MAF	0.031505	1.032007	0.03036
ABCA1	0.081281	1.084676	0.000577
NTAN1	0.127768	1.136289	0.000319
CD59	0.027812	1.028202	0.000719
GUSB	0.01137	1.011435	0.039202
ENG	0.009902	1.009951	0.00234
AGPAT2	-0.00504	0.994971	0.012344
CCPG1	0.224044	1.251127	0.009341
FBXO6	-0.03152	0.968971	0.009159
SLC35F6	-0.02741	0.972963	0.023458
MRPS25	-0.09763	0.906989	0.030507
PLXNC1	0.119731	1.127194	0.000609
PTTG1IP	0.008179	1.008213	0.009696
CD302	0.118299	1.12558	0.011058
DPP9	-0.05494	0.946542	0.017841

B, regression coefficient; HR, hazard ratio.

**Table 2:** Multivariate Cox regression analysis of high-OXPHOS-macrophage related genes.

Gene	B	HR	*P*-value
NRP1	0.075608	1.07854	0.0002
C1orf54	-0.0768	0.926076	0.000536
NPC2	-0.01168	0.988391	0.000426
RNASE1	0.000686	1.000686	0.000588
AKR1B1	0.018139	1.018305	0.000513
LY96	0.020804	1.021022	0.000286
TPP1	0.010523	1.010578	0.000498
CALU	-0.01271	0.987375	0.00026
GSN	-0.00853	0.991507	0.000303
CPNE8	0.113155	1.119806	0.000116
ELOVL1	-0.01131	0.98875	0.000571
BEX4	0.017917	1.018078	0.000849
NTAN1	0.151045	1.163049	0.000608
CD59	0.025899	1.026237	0.000497
GUSB	0.017206	1.017355	0.000985
FBXO6	-0.02942	0.971012	0.000592
MRPS25	0.08982	1.093977	0.000441
PTTG1IP	0.008328	1.008362	0.000733
DPP9	-0.0391	0.961659	0.000301

B, regression coefficient; HR, hazard ratio.

### Expression pattern of high-OXPHOS-macrophage related prognostic signature

**Figure 4:**
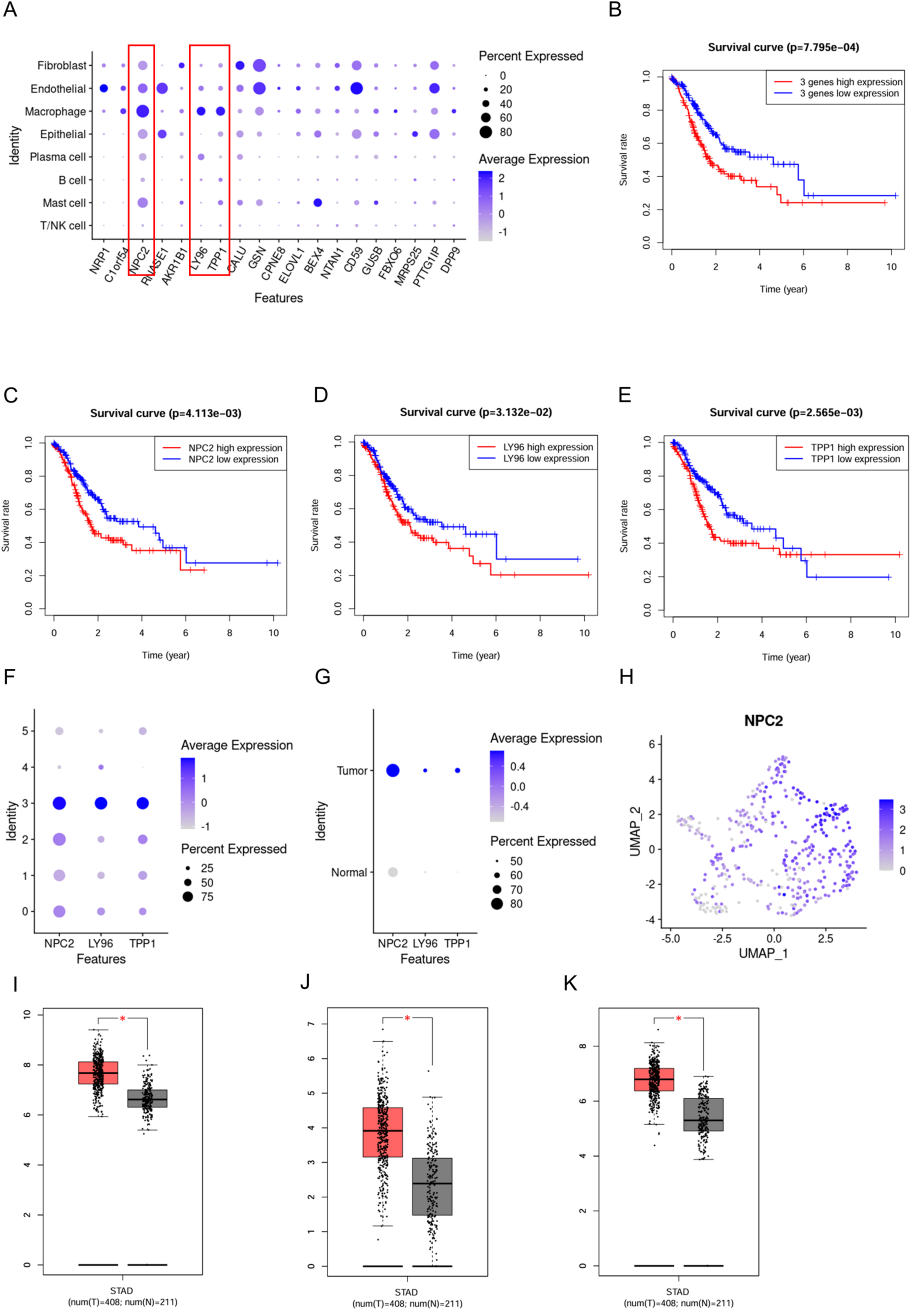
Expression pattern of the prognostic signature. **(A)** Expression distribution of the prognostic signature in different cell types,where blue dots represent high expression levels, and gray dots represent low expression levels. **(B)** Survival curve for 3 prognostic genes. **(C)** Survival curve for NPC2. **(D)** Survival curve for LY96. **(E)** Survival curve for TPP1. **(F)** Expression distribution of the NPC2, LY96 and TPP1 in macrophage subgroups. **(G)** Expression distribution of the NPC2, LY96 and TPP1 in TAMs and normal macrophages. **(H)** Expression distribution of the NPC2 in all macrophages. **(I)** Box plot for NPC2. **(J)** Box plot for LY96. **(K)** Box plot for TPP1. *P < 0.05.

The 19 genes that make up the high-OXPHOS-macrophage related prognostic signature are all expressed in macrophages, however, NPC2, LY96, and TPP1 are specifically expressed in macrophages (**Figure 4A**). Among these, patients with high expression of NPC2, LY96, and TPP1 have a poorer prognosis (**Figures 4B–E**). In macrophages, NPC2, LY96, and TPP1 are mainly expressed in type C3 macrophages, and the expression level of NPC2 is higher than that of LY96 and TPP1 (**Figure 4F**). Moreover, NPC2, LY96, and TPP1 are primarily highly expressed in TAMs (**Figure 4G**), and the expression level of NPC2 is significantly higher than LY96 and TPP1 (**Figure 4H**). This demonstrates that NPC2 plays an important role in TAMs. At the single-cell level, NPC2, LY96, and TPP1 are significantly elevated in TAMs. Data from TCGA were used to explore the expression differences of NPC2, LY96, and TPP1 at the overall tumor level. Ultimately, we found that NPC2, LY96, and TPP1 are highly expressed in tumor tissues (**Figures 4I–K**). NPC2, LY96, and TPP1 were most likely related to the function of TAM.

### The impact of NPC2 positive macrophages on the tumor microenvironment

**Figure 5:**
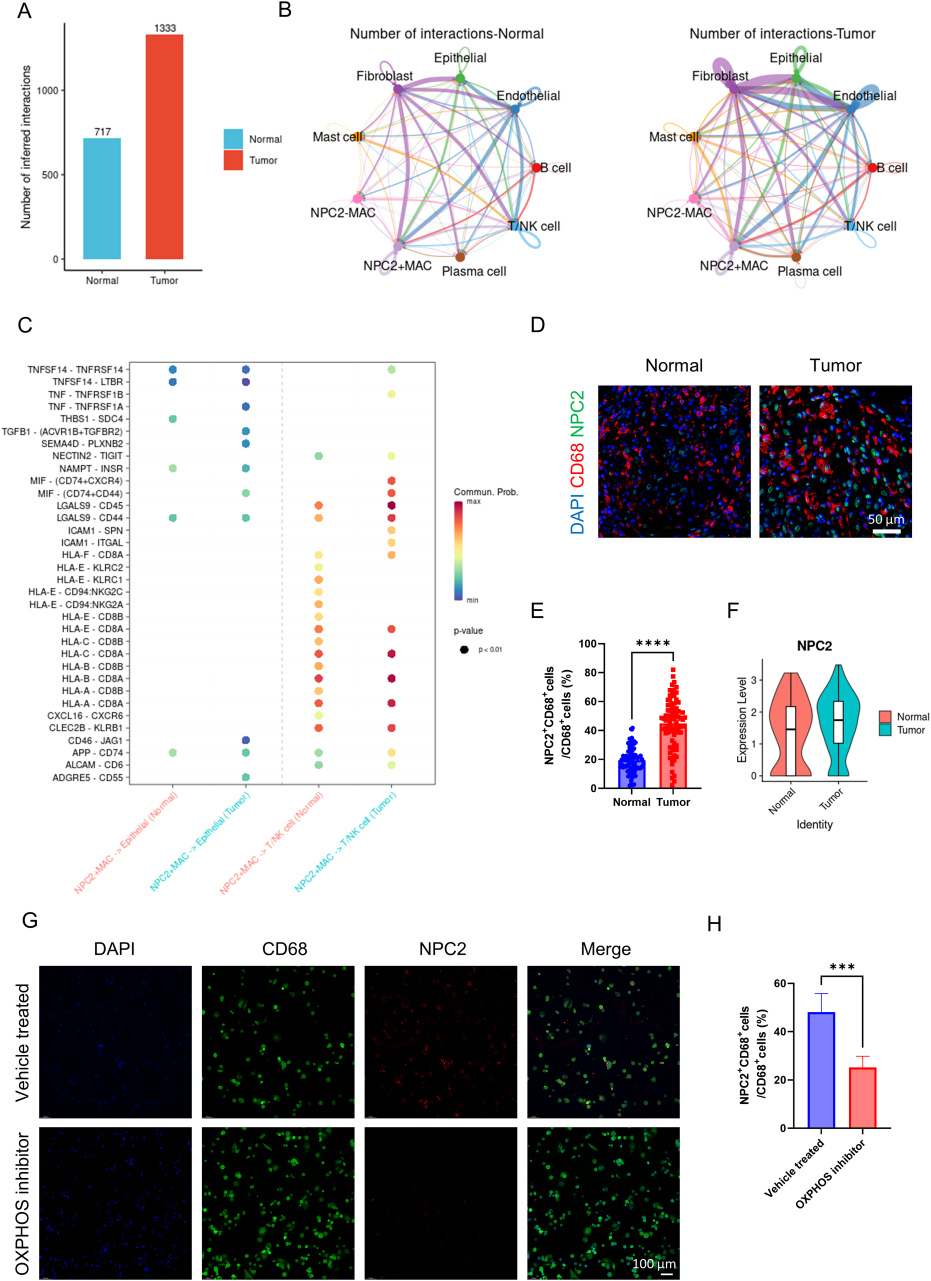
Analysis of NPC2 related cell communication. **(A)** the intensity of cell communication in tumor tissue is higher than that in normal tissue. **(B)** the intensity of communication between cells in tumor tissue and in normal tissue. **(C)** NPC2-positive macrophages, tumor cells and T cells/NK cells ligand-receptor pairs, with red indicating increased activity of ligand-receptor pairs and blue indicating decreased activity. **(D)** representative immunofluorescence images of NPC2 expression in macrophages in tumor and normal tissues. Scale bar: 50 μm (40X). **(E)** quantification of immunofluorescence (n = 80 each group). **(F)** NPC2 expression level of macrophages from tumor and normal group (external validation). **(G)** representative immunofluorescence images of NPC2 expression in macrophages in vehicle treated and OXPHOS inhibitor groups. Scale bar: 100 μm (40X). **(H)** quantification of immunofluorescence (n = 3 each group). ***P <0.001, ****P <0.0001.

We classified macrophages into NPC2 positive macrophages and NPC2 negative macrophages based on the expression level of NPC2. When observing the overall communication strength of cells, cell communication in tumor tissues was significantly stronger than in normal tissues (**Figure 5A**). The communication strength between different cell types in tumor tissues was clearly stronger than that between cell types in normal tissues (**Figure 5B**). NPC2 positive macrophages secrete TNF and TGFB1 that act on tumor cells, thereby enhancing the proliferation of tumor cells (**Figure 5C**). Furthermore, research has confirmed that MIF acts on T cells, causing the suppression of T cell activity and thereby mediating tumor immune escape. Similarly, NPC2 positive macrophages secrete MIF acting on T cells, inhibiting T cell activity and mediating tumor immune escape (**Figure 5C**). The above results indicate that NPC2 positive macrophages can affect the tumor microenvironment, ultimately promoting tumor growth and invasion. Lastly, we confirmed the high expression of NPC2 in tumor tissue macrophages using immunofluorescence (**Figures 5D, E**). Through external validation using GSE268238, we confirmed that NPC2 was highly expressed in macrophages from tumor tissues (**Figure 5F**). After using the OXPHOS inhibitor, we found that the expression of NPC2 in macrophages was significantly reduced (**Figures 5G, H**). This further illustrates the close relationship between NPC2 and OXPHOS.

### The impact of LY96 positive macrophages on the tumor microenvironment

**Figure 6:**
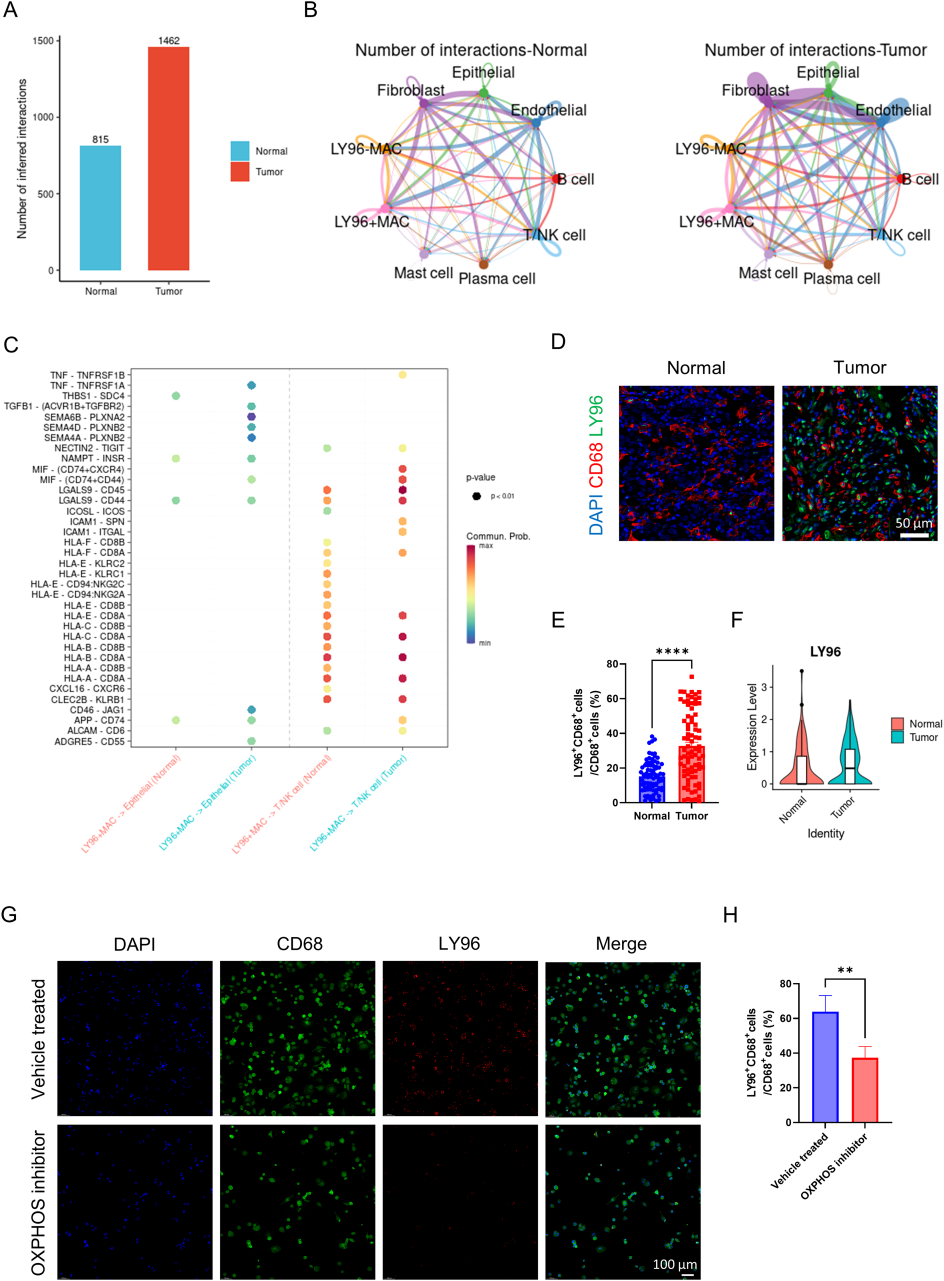
Analysis of LY96 related cell communication. **(A)** the intensity of cell communication in tumor tissue is higher than that in normal tissue. **(B)** the intensity of communication between cells in tumor tissue and in normal tissue. **(C)** LY96-positive macrophages, tumor cells and T cells/NK cells ligand-receptor pairs, with red indicating increased activity of ligand-receptor pairs and blue indicating decreased activity. **(D)** representative immunofluorescence images of LY96 expression in macrophages in tumor and normal tissues. Scale bar: 50 μm (40X). **(E)** quantification of immunofluorescence (n = 80 each group). **(F)** LY96 expression level of macrophages from tumor and normal group (external validation). **(G)** representative immunofluorescence images of LY96 expression in macrophages in vehicle treated and OXPHOS inhibitor groups. Scale bar: 100 μm (40X). **(H)** quantification of immunofluorescence (n = 3 each group). **P <0.01, ****P <0.0001.

We classified macrophages into LY96 positive macrophages and LY96 negative macrophages based on the expression level of LY96. The overall cell communication strength is significantly stronger in tumor tissues compared to normal tissues (**Figure 6A**). The communication strength among different cell types within tumor tissues is significantly stronger than that within normal tissues (**Figure 6B**). Similar to before, LY96 positive macrophages secrete TNF and TGFB1 that act on tumor cells, thereby enhancing tumor cell proliferation (**Figure 6C**). Like NPC2, LY96 positive macrophages secrete MIF affecting T cells, inhibiting T cell activity, and mediating tumor immune escape (**Figure 6C**). These results demonstrate that LY96 positive macrophages can influence the tumor microenvironment, ultimately promoting tumor growth and invasion. Finally, we confirmed the high expression of LY96 in tumor tissue macrophages using immunofluorescence (**Figures 6D, E**). Through external validation using GSE268238, we confirmed that LY96 was highly expressed in macrophages from tumor tissues (**Figure 6F**). After using the OXPHOS inhibitor, we found that the expression of LY96 in macrophages was significantly reduced (**Figures 6G, H**). This further illustrates the close relationship between LY96 and OXPHOS.

### The impact of TPP1 positive macrophages on the tumor microenvironment

**Figure 7:**
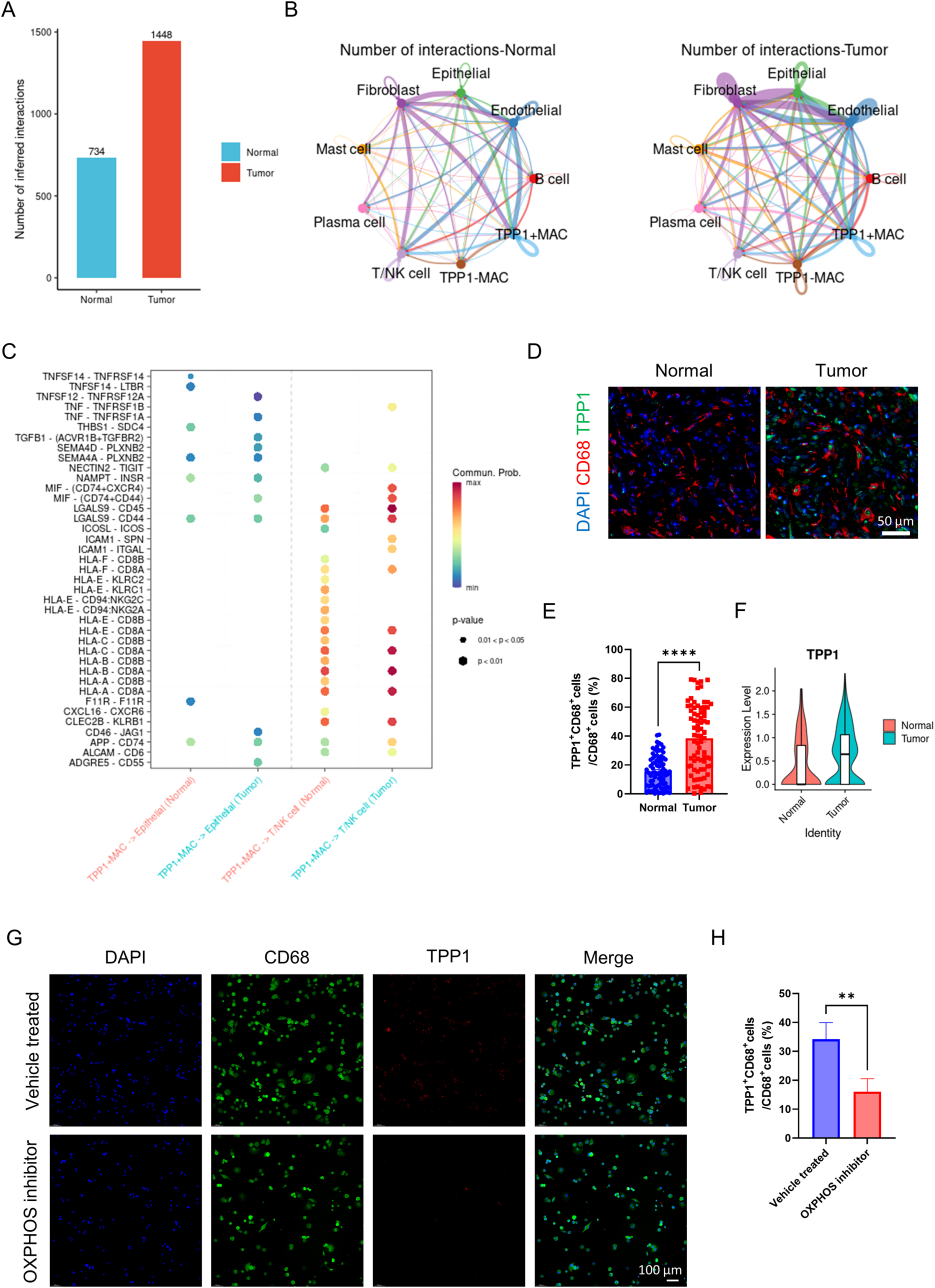
Analysis of TPP1 related cell communication. **(A)** the intensity of cell communication in tumor tissue is higher than that in normal tissue. **(B)** the intensity of communication between cells in tumor tissue and in normal tissue. **(C)** TPP1-positive macrophages, tumor cells and T cells/NK cells ligand-receptor pairs, with red indicating increased activity of ligand-receptor pairs and blue indicating decreased activity. **(D)** representative immunofluorescence images of TPP1 expression in macrophages in tumor and normal tissues. Scale bar: 50 μm (40X). **(E)** quantification of immunofluorescence (n = 80 each group). **(F)** TPP1 expression level of macrophages from tumor and normal group (external validation). **(G)** representative immunofluorescence images of TPP1 expression in macrophages in vehicle treated and OXPHOS inhibitor groups. Scale bar: 100 μm (40X). **(H)** quantification of immunofluorescence (n = 3 each group). **P <0.01, ****P <0.0001.

We classified macrophages into TPP1 positive macrophages and TPP1 negative macrophages based on the expression level of TPP1. The overall cell communication strength in tumor tissues was notably stronger than in normal tissues (**Figure 7A**). Communication strength among different cell types within tumor tissues was clearly stronger than that within normal tissues (**Figure 7B**). Similar to the previous findings, TPP1 positive macrophages secrete TNF and TGFB1 acting on tumor cells, thereby enhancing the proliferation of tumor cells (**Figure 7C**). In a resembling manner, TPP1 positive macrophages secrete MIF acting on T cells, thus inhibiting T cell activity and mediating tumor immune escape (**Figure 7C**). The aforementioned results suggest that TPP1 positive macrophages can affect the tumor microenvironment, leading to the promotion of tumor growth and invasion. Lastly, using immunofluorescence, we verified the high expression of TPP1 in the macrophages of tumor tissue (**Figures 7D, E**). Through external validation using GSE268238, we confirmed that TPP1 was highly expressed in macrophages from tumor tissues (**Figure 7F**). After using the OXPHOS inhibitor, we found that the expression of TPP1 in macrophages was significantly reduced (**Figures 7G, H**). This further illustrates the close relationship between TPP1 and OXPHOS.

## Discussion

Our comprehensive single-cell sequencing analysis elucidated the transcriptomic landscape of GC and the surroundings of its microenvironment, revealing crucial cellular heterogeneity and multiple significant insights into the role of macrophages in gastric carcinogenesis. Of particular note, our findings underscored the involvement of macrophages with high oxidative phosphorylation activity and their associated genes in GC prognosis.

In this study, we identified distinct cell populations within the tumor microenvironment, with TAMs standing out due to their substantial presence in tumor tissues. The elevated proportion of TAMs, particularly the C3 subgroup, bearing high OXPHOS scores, hints at their potential contribution to tumorigenesis via creation of an environment conducive to tumor growth and invasion. This inference falls in line with prior research emphasizing the tumor-promoting nature of M2-like TAMs characterized by their metabolic reprogramming (29, 30).

Importantly, our prognostic signature, which was derived from high-OXPHOS-macrophage-related genes, serves as a robust tool with significant stratification power for GC prognosis. This is evident from our Kaplan-Meier survival analyses and the high AUC in validation using the TCGA dataset. Consequently, it provides a novel angle from which to assess risk factors and stratify patients, potentially leading to more tailored therapeutic interventions.

Our scrutiny of the prognostic signature revealed that among the integral genes, NPC2, LY96, and TPP1, all primarily expressed in macrophages, notably the C3 subtype, are of exceptional interest. Their high expression within GC tissues was linked to a poorer prognosis. This association could be attributed to the observed intensified cell communication within tumor tissues, particularly the interactions mediated by these macrophage-related genes that appear to promote cell proliferation and facilitate immune evasion.

Indeed, the categorization of macrophages according to NPC2, LY96, and TPP1 expression highlighted their differential impact on the tumor microenvironment. These gene-positive macrophages secreted factors such as TNF, TGFB1, and notably MIF, which is implicated in T cell suppression—suggesting a complex role of these macrophages in both propagating tumor growth as well as modulating the immune landscape to favor tumor immune escape (31).

The higher cell communication strength visible in tumor tissues validates the hypothesis that the intricate network of cross-talk among various cell types contributes to the complex pathophysiology of GC. Intriguingly, the higher expression of NPC2, LY96, and TPP1 could be a marker of such intensive communication, enabling these macrophages to significantly influence the microenvironment.

Confirmatory immunofluorescence staining affirmed the high presence of these markers in GC tissues, lending credence to the idea that their expression can be visualized and potentially targeted *in situ*. While these observations offer valuable prognostic information, they may also pave the way for therapeutic innovations; targeting these macrophage populations or disrupting their communication with tumor cells might represent a novel strategy in combatting GC.

The present study, however, is not without limitations. Despite the robust bioinformatic and statistical analysis, functional experiments in *in vitro* and *in vivo* models are necessary to validate the causal roles of the identified genes and macrophage subsets in GC progression. Furthermore, therapeutic trials will be essential to test whether manipulation of these gene-related pathways can effectively treat GC.

In conclusion, our study highlights the significance of macrophages with elevated OXPHOS activity in the gastric cancer microenvironment and unveils a prognostic signature that could improve patient stratification and targeting of GC therapy. This lays the groundwork for future research aiming to translate these biomarkers into clinical practice and to investigate potential therapies targeting these pathways.

## Data Availability

Publicly available datasets were analyzed in this study. This data can be found here: The sequencing data was available in GEO (GSE184198, GSE62254).
