# Supplementary material for: Prognostic implications of high- OXPHOS macrophages in gastric cancer: a single-cell transcriptomics and tumor microenvironment communication study
Source: Front Oncol. 2025 Jun 20;15:1533990. doi: 10.3389/fonc.2025.1533990 (PMC12226282; doi:10.3389/fonc.2025.1533990)
Supplement: Supplementary file 1 [file Table1.docx]

**Table S1 The genes of OXPHOS score calculation**

| Gene |
| --- |
| UQCRFS1P1 |
| NDUFC2-KCTD14 |
| PPIF |
| ATP5PD |
| ATP5MG |
| MTCO2P12 |
| UQCR11 |
| PARK7 |
| COX6B2 |
| NDUFA11 |
| COX4I1 |
| COX5B |
| COX6A1 |
| COX6A2 |
| COX6B1 |
| ATPSCKMT |
| COX6C |
| COX7A1 |
| COX7A2 |
| COX7A2P2 |
| COX7B |
| COX7C |
| COX8A |
| CYC1 |
| COX7B2 |
| DGUOK |
| DLD |
| AK4 |
| C2orf69 |
| ABCD1 |
| ISCU |
| FXN |
| AFG1L |
| NIPSNAP2 |
| NUPR1 |
| GHITM |
| UQCRQ |
| DNAJC15 |
| UQCR10 |
| STOML2 |
| COX8C |
| NDUFS7 |
| RHOA |
| COA6 |
| CHCHD10 |
| MIR210 |
| ATP5F1EP2 |
| UQCRHL |
| MSH2 |
| MT-ATP6 |
| MT-ATP8 |
| MT-CO1 |
| MT-CO2 |
| MT-CO3 |
| MT-CYB |
| MT-ND1 |
| MT-ND2 |
| MT-ND3 |
| MT-ND4 |
| MT-ND4L |
| MT-ND5 |
| MT-ND6 |
| NDUFA1 |
| NDUFA2 |
| NDUFA3 |
| NDUFA4 |
| NDUFA5 |
| NDUFA6 |
| NDUFA7 |
| NDUFA8 |
| NDUFA9 |
| NDUFA10 |
| NDUFAB1 |
| NDUFB1 |
| NDUFB2 |
| NDUFB3 |
| NDUFB4 |
| NDUFB5 |
| NDUFB6 |
| NDUFB7 |
| NDUFB8 |
| NDUFB9 |
| NDUFB10 |
| NDUFC1 |
| NDUFC2 |
| NDUFS1 |
| NDUFS2 |
| NDUFS3 |
| NDUFV1 |
| NDUFS4 |
| NDUFS5 |
| NDUFS6 |
| NDUFS8 |
| NDUFV2 |
| NDUFV3 |
| ATP5F1A |
| ATP5F1B |
| ATP5F1C |
| NDUFA13 |
| MLXIPL |
| NDUFAF1 |
| CHCHD2 |
| ATP5F1D |
| ATP5F1E |
| ATP5PB |
| ATP5ME |
| ATP5PF |
| ATP7A |
| ATP5PO |
| CYCS |
| NDUFB11 |
| SDHAF2 |
| NDUFA12 |
| COQ9 |
| BID |
| SDHA |
| SDHB |
| SDHC |
| SDHD |
| SHMT2 |
| PINK1 |
| TMEM135 |
| ANTKMT |
| SNCA |
| TAFAZZIN |
| TNF |
| UQCRB |
| UQCRC1 |
| UQCRC2 |
| UQCRFS1 |
| UQCRH |
| VCP |
| SLC25A23 |
| UQCC3 |
| TEFM |
| SLC25A33 |
| DNAJC30 |
| UQCC2 |
| COX4I2 |
| ACTN3 |
| CCNB1 |
| COX7A2L |
| SLC25A51 |
| COX5A |
| ATP5MF |
| MACROH2A1 |
| CDK1 |

**Table S2 The OXPHOS scores of each macrophage**

| Cell ID | seurat_clusters | cell_type | group | OXPHOS_Score |
| --- | --- | --- | --- | --- |
| N_AACAGGGAGTTGGCGA-1 | 4 | Macrophage | Normal | 0.547720583 |
| N_AAGTACCAGGCACTCC-1 | 2 | Macrophage | Normal | 0.365543805 |
| N_AATGGCTTCGCCGAAC-1 | 4 | Macrophage | Normal | 0.472456676 |
| N_ACAACCAAGGGCAACT-1 | 2 | Macrophage | Normal | 0.458877026 |
| N_ACACCAAGTCTCGACG-1 | 2 | Macrophage | Normal | 0.347735997 |
| N_ACGATCAAGGCTATCT-1 | 4 | Macrophage | Normal | 0.524564793 |
| N_ACGTACATCGTGCATA-1 | 2 | Macrophage | Normal | 0.539619332 |
| N_ACGTTCCCAGGTATGG-1 | 2 | Macrophage | Normal | 0.379926317 |
| N_ACTATGGAGGCGTCCT-1 | 2 | Macrophage | Normal | 0.500426277 |
| N_ACTTTCACAACGGCCT-1 | 1 | Macrophage | Normal | 0.354860251 |
| N_AGAACCTCACAAGGTG-1 | 2 | Macrophage | Normal | 0.325268837 |
| N_AGAAGTAAGGAGACCT-1 | 2 | Macrophage | Normal | 0.257023419 |
| N_AGACAAATCGCTTTAT-1 | 2 | Macrophage | Normal | 0.308709501 |
| N_AGGGTTTGTACGTAGG-1 | 1 | Macrophage | Normal | 0.596789519 |
| N_AGTAGCTAGCTAGCCC-1 | 5 | Macrophage | Normal | 0.466855274 |
| N_AGTTAGCGTACTCCCT-1 | 2 | Macrophage | Normal | 0.445005124 |
| N_ATATCCTAGTGCAAAT-1 | 4 | Macrophage | Normal | 0.478389071 |
| N_ATATCCTGTGCTGCAC-1 | 2 | Macrophage | Normal | 0.40580686 |
| N_ATCACAGTCCGTGGTG-1 | 3 | Macrophage | Normal | 0.505966333 |
| N_ATCACTTTCATTGAGC-1 | 2 | Macrophage | Normal | 0.340357571 |
| N_ATCCGTCCAGATTTCG-1 | 4 | Macrophage | Normal | 0.482952961 |
| N_ATCGATGCAGCTGGTC-1 | 4 | Macrophage | Normal | 0.380007792 |
| N_ATCGATGTCTACGGGC-1 | 2 | Macrophage | Normal | 0.560296282 |
| N_ATCGGATAGGAAAGTG-1 | 2 | Macrophage | Normal | 0.430727068 |
| N_ATTCATCTCACTCCGT-1 | 2 | Macrophage | Normal | 0.592562196 |
| N_ATTCCATTCAGCCTTC-1 | 4 | Macrophage | Normal | 0.319941484 |
| N_CACGAATTCCCATACC-1 | 4 | Macrophage | Normal | 0.506519516 |
| N_CATACCCGTCTGCATA-1 | 2 | Macrophage | Normal | 0.58318038 |
| N_CATCCACTCTATCGGA-1 | 2 | Macrophage | Normal | 0.407889572 |
| N_CATTGAGTCGTCCATC-1 | 0 | Macrophage | Normal | 0.454089303 |
| N_CATTGCCGTGACACGA-1 | 0 | Macrophage | Normal | 0.721922466 |
| N_CCACGAGTCATTGAGC-1 | 0 | Macrophage | Normal | 0.307520782 |
| N_CCCGGAAAGAAGGGAT-1 | 1 | Macrophage | Normal | 0.343276284 |
| N_CCTTGTGGTCAGACTT-1 | 2 | Macrophage | Normal | 0.302343416 |
| N_CGGACACGTCGAGATG-1 | 1 | Macrophage | Normal | 0.41670524 |
| N_CGGGCATTCGACCACG-1 | 2 | Macrophage | Normal | 0.445930232 |
| N_CGTGCTTGTCGAGCAA-1 | 4 | Macrophage | Normal | 0.500921128 |
| N_CGTTCTGCACGGTGAA-1 | 2 | Macrophage | Normal | 0.44609034 |
| N_CTAGGTAGTGAGCGAT-1 | 4 | Macrophage | Normal | 0.301448257 |
| N_CTCATCGGTCCAAGAG-1 | 4 | Macrophage | Normal | 0.502565564 |
| N_CTCTGGTAGTAAGGGA-1 | 1 | Macrophage | Normal | 0.657363019 |
| N_CTGAGGCGTTACGGAG-1 | 4 | Macrophage | Normal | 0.495909083 |
| N_CTGCAGGGTTGCGAAG-1 | 2 | Macrophage | Normal | 0.490525648 |
| N_GAGATGGTCTCCTGCA-1 | 2 | Macrophage | Normal | 0.359957849 |
| N_GAGTGAGAGTGGGAAA-1 | 2 | Macrophage | Normal | 0.539895495 |
| N_GCATGATTCGACTCCT-1 | 2 | Macrophage | Normal | 0.430883893 |
| N_GCCAGTGTCCACACAA-1 | 4 | Macrophage | Normal | 0.400739451 |
| N_GCCCAGACAACGGCTC-1 | 4 | Macrophage | Normal | 0.460785333 |
| N_GCGATCGTCCCTAGGG-1 | 2 | Macrophage | Normal | 0.516195574 |
| N_GGCTTTCTCAATCAGC-1 | 2 | Macrophage | Normal | 0.237864091 |
| N_GGGAGATGTCTATGAC-1 | 2 | Macrophage | Normal | 0.522327109 |
| N_GGGTCACAGAATAGTC-1 | 4 | Macrophage | Normal | 0.457033945 |
| N_GGGTCTGGTATCTCGA-1 | 4 | Macrophage | Normal | 0.505184932 |
| N_GGGTTTATCTTCCCGA-1 | 2 | Macrophage | Normal | 0.367158975 |
| N_GGTGATTAGGTCGTCC-1 | 4 | Macrophage | Normal | 0.413423318 |
| N_GTACAACGTACCTGTA-1 | 0 | Macrophage | Normal | 0.847398046 |
| N_GTCAAGTAGCGATGAC-1 | 2 | Macrophage | Normal | 0.472640882 |
| N_GTCAGCGGTAGCTTGT-1 | 2 | Macrophage | Normal | 0.447072343 |
| N_GTCAGCGGTTAGGCTT-1 | 2 | Macrophage | Normal | 0.525347066 |
| N_GTGCTGGTCGCTTAAG-1 | 4 | Macrophage | Normal | 0.328319204 |
| N_GTGGAGAGTGTCCCTT-1 | 2 | Macrophage | Normal | 0.588654133 |
| N_GTGGAGATCTTCCACG-1 | 2 | Macrophage | Normal | 0.49487067 |
| N_GTTACCCTCATGACAC-1 | 4 | Macrophage | Normal | 0.468560806 |
| N_GTTGAACAGAACCGCA-1 | 0 | Macrophage | Normal | 0.364135983 |
| N_TACCTCGTCCACTTTA-1 | 2 | Macrophage | Normal | 0.341236406 |
| N_TACGGGCCAAAGGTTA-1 | 2 | Macrophage | Normal | 0.460564553 |
| N_TACTTGTCAACGTTAC-1 | 2 | Macrophage | Normal | 0.460754768 |
| N_TATCTTGCAACAACAA-1 | 4 | Macrophage | Normal | 0.369606096 |
| N_TCAATCTTCAAACTGC-1 | 4 | Macrophage | Normal | 0.401881495 |
| N_TCACGGGCAGATCACT-1 | 4 | Macrophage | Normal | 0.437380761 |
| N_TCACGGGTCTACTGAG-1 | 1 | Macrophage | Normal | 0.41715841 |
| N_TCAGTGACACGTCATA-1 | 1 | Macrophage | Normal | 0.633803802 |
| N_TCCCACAAGTAACCGG-1 | 3 | Macrophage | Normal | 0.555365235 |
| N_TCCCATGCATGCAGCC-1 | 2 | Macrophage | Normal | 0.496610205 |
| N_TCCTTTCAGAACGCGT-1 | 4 | Macrophage | Normal | 0.426594377 |
| N_TGCCGAGTCACAGAGG-1 | 3 | Macrophage | Normal | 0.523829703 |
| N_TGCGACGAGGAGAGGC-1 | 2 | Macrophage | Normal | 0.351932954 |
| N_TGCTGAACAAAGAACT-1 | 1 | Macrophage | Normal | 0.449335973 |
| N_TGGTTAGAGTGGATTA-1 | 2 | Macrophage | Normal | 0.219563015 |
| N_TGTGGCGTCTCGACGG-1 | 2 | Macrophage | Normal | 0.529166735 |
| N_TTATTGCTCGAGAAAT-1 | 2 | Macrophage | Normal | 0.395825452 |
| N_TTGCCTGAGGTCCGAA-1 | 0 | Macrophage | Normal | 0.266387028 |
| N_TTGTTTGTCTTTGCGC-1 | 1 | Macrophage | Normal | 0.398920743 |
| N_TTTGATCAGTAAACTG-1 | 2 | Macrophage | Normal | 0.461389465 |
| T_AAACGCTCACGGATCC-1 | 5 | Macrophage | Tumor | 0.300728517 |
| T_AAAGAACAGGATTTCC-1 | 1 | Macrophage | Tumor | 0.491720389 |
| T_AAAGGTAAGACCATGG-1 | 0 | Macrophage | Tumor | 0.383980111 |
| T_AAAGTGATCGTTGTTT-1 | 0 | Macrophage | Tumor | 0.477921349 |
| T_AACAGGGAGATAACAC-1 | 5 | Macrophage | Tumor | 0.62414637 |
| T_AACCACAAGGTTTGAA-1 | 3 | Macrophage | Tumor | 0.659420589 |
| T_AACCTGATCCTTCAGC-1 | 0 | Macrophage | Tumor | 0.439227342 |
| T_AACGGGACAGGAACCA-1 | 0 | Macrophage | Tumor | 0.560791905 |
| T_AACGGGATCGGAACTT-1 | 1 | Macrophage | Tumor | 0.326439897 |
| T_AAGAACAAGAGGGCGA-1 | 4 | Macrophage | Tumor | 0.462618705 |
| T_AAGCATCAGGGACACT-1 | 0 | Macrophage | Tumor | 0.360507186 |
| T_AAGGAATTCCATGATG-1 | 3 | Macrophage | Tumor | 0.621201212 |
| T_AAGTCGTGTGCCGAAA-1 | 0 | Macrophage | Tumor | 0.515311922 |
| T_AATGGAAGTCGTTTCC-1 | 1 | Macrophage | Tumor | 0.267488457 |
| T_ACACCAACAGTGCGCT-1 | 0 | Macrophage | Tumor | 0.492790071 |
| T_ACAGCCGGTCTTGTCC-1 | 3 | Macrophage | Tumor | 0.694067719 |
| T_ACAGGGACAGCTACTA-1 | 0 | Macrophage | Tumor | 0.532817965 |
| T_ACAGGGAGTAGCTTTG-1 | 1 | Macrophage | Tumor | 0.4462326 |
| T_ACATGCAGTAGGCTCC-1 | 1 | Macrophage | Tumor | 0.491433363 |
| T_ACGGAAGCAGCAGAAC-1 | 0 | Macrophage | Tumor | 0.471502396 |
| T_ACGTAACCATGCACTA-1 | 0 | Macrophage | Tumor | 0.398635011 |
| T_ACTACGAAGGTTCCAT-1 | 1 | Macrophage | Tumor | 0.389387992 |
| T_ACTGCAACAGCTACTA-1 | 1 | Macrophage | Tumor | 0.357796695 |
| T_ACTGTCCAGGTTGACG-1 | 5 | Macrophage | Tumor | 0.442429766 |
| T_ACTGTCCGTTAGCTAC-1 | 1 | Macrophage | Tumor | 0.444181892 |
| T_ACTTAGGGTCACTACA-1 | 1 | Macrophage | Tumor | 0.458501306 |
| T_ACTTCCGGTCGCTTGG-1 | 0 | Macrophage | Tumor | 0.53571085 |
| T_ACTTCCGGTGACCGTC-1 | 4 | Macrophage | Tumor | 0.363556955 |
| T_AGAACCTAGATTGGGC-1 | 0 | Macrophage | Tumor | 0.320085625 |
| T_AGACAGGTCTAGCATG-1 | 5 | Macrophage | Tumor | 0.603402818 |
| T_AGACTCAAGGTTGGTG-1 | 1 | Macrophage | Tumor | 0.467578464 |
| T_AGACTCAAGTTGCTCA-1 | 1 | Macrophage | Tumor | 0.306845812 |
| T_AGATGCTCAAAGGGCT-1 | 1 | Macrophage | Tumor | 0.354358913 |
| T_AGCCAATTCACTTCTA-1 | 0 | Macrophage | Tumor | 0.409896741 |
| T_AGCCACGGTTTGATCG-1 | 0 | Macrophage | Tumor | 0.408081982 |
| T_AGCGCCATCCATCACC-1 | 1 | Macrophage | Tumor | 0.584156314 |
| T_AGCGCTGAGTTAACGA-1 | 0 | Macrophage | Tumor | 0.304574081 |
| T_AGCGCTGTCGCCTTGT-1 | 0 | Macrophage | Tumor | 0.419631047 |
| T_AGCTCAACACAACGAG-1 | 3 | Macrophage | Tumor | 0.621819696 |
| T_AGGACGAAGATTTGCC-1 | 1 | Macrophage | Tumor | 0.57064452 |
| T_AGGCATTGTGTTGAGG-1 | 0 | Macrophage | Tumor | 0.487576327 |
| T_AGGGCCTAGTCATAGA-1 | 5 | Macrophage | Tumor | 0.206180992 |
| T_AGGTAGGAGCGACAGT-1 | 4 | Macrophage | Tumor | 0.344669779 |
| T_AGGTTGTAGGGTTAGC-1 | 3 | Macrophage | Tumor | 0.499193315 |
| T_AGTCATGGTCTGATCA-1 | 0 | Macrophage | Tumor | 0.427161753 |
| T_AGTGTTGAGGCGCTCT-1 | 0 | Macrophage | Tumor | 0.481638611 |
| T_ATACCTTGTTGAGTCT-1 | 0 | Macrophage | Tumor | 0.449482588 |
| T_ATATCCTAGAATCGTA-1 | 0 | Macrophage | Tumor | 0.303291173 |
| T_ATCATTCTCGCTATTT-1 | 0 | Macrophage | Tumor | 0.517052766 |
| T_ATCCACCCAGCTGCCA-1 | 0 | Macrophage | Tumor | 0.544252057 |
| T_ATCCCTGAGTTCATCG-1 | 0 | Macrophage | Tumor | 0.455769049 |
| T_ATCCGTCCAGACCCGT-1 | 0 | Macrophage | Tumor | 0.339921279 |
| T_ATCCGTCTCCACTTTA-1 | 0 | Macrophage | Tumor | 0.317129013 |
| T_ATCCTATTCGATGCAT-1 | 1 | Macrophage | Tumor | 0.588232627 |
| T_ATCGTCCGTTCCTAAG-1 | 3 | Macrophage | Tumor | 0.415402183 |
| T_ATCTCTAGTGCACGCT-1 | 3 | Macrophage | Tumor | 0.584079087 |
| T_ATGAGGGTCTACTCAT-1 | 0 | Macrophage | Tumor | 0.309858237 |
| T_ATGAGTCTCGCTTTAT-1 | 5 | Macrophage | Tumor | 0.388612218 |
| T_ATGATCGCAGGCATGA-1 | 1 | Macrophage | Tumor | 0.537859947 |
| T_ATGGAGGCACTCCTGT-1 | 1 | Macrophage | Tumor | 0.528038559 |
| T_ATGGATCAGAAGAACG-1 | 0 | Macrophage | Tumor | 0.353937894 |
| T_ATGGTTGAGTTTCGAC-1 | 4 | Macrophage | Tumor | 0.424611883 |
| T_ATTCCCGAGATGCTAA-1 | 4 | Macrophage | Tumor | 0.427506613 |
| T_ATTCCCGCATGAGTAA-1 | 3 | Macrophage | Tumor | 0.361185112 |
| T_ATTTCACAGGAACGTC-1 | 1 | Macrophage | Tumor | 0.584543461 |
| T_ATTTCACGTCGTTTCC-1 | 5 | Macrophage | Tumor | 0.53259663 |
| T_CAAAGAAGTACGCGTC-1 | 0 | Macrophage | Tumor | 0.479846554 |
| T_CAACAGTGTCACGACC-1 | 3 | Macrophage | Tumor | 0.558349277 |
| T_CAACCTCGTCGCACGT-1 | 0 | Macrophage | Tumor | 0.252683936 |
| T_CAACGATAGTAGAATC-1 | 0 | Macrophage | Tumor | 0.482818468 |
| T_CAACGGCCATGCCGAC-1 | 3 | Macrophage | Tumor | 0.325920984 |
| T_CAAGACTGTATAATGG-1 | 1 | Macrophage | Tumor | 0.400766774 |
| T_CAATACGAGGTGCCTC-1 | 0 | Macrophage | Tumor | 0.507325195 |
| T_CAATGACTCTCGCCTA-1 | 0 | Macrophage | Tumor | 0.543841047 |
| T_CACCAAAGTAAGATTG-1 | 0 | Macrophage | Tumor | 0.573361934 |
| T_CACTGTCAGGAATTAC-1 | 0 | Macrophage | Tumor | 0.454294475 |
| T_CACTGTCGTCGTGGTC-1 | 0 | Macrophage | Tumor | 0.352732698 |
| T_CACTGTCTCATCTATC-1 | 0 | Macrophage | Tumor | 0.391643836 |
| T_CACTTCGGTTCTATCT-1 | 0 | Macrophage | Tumor | 0.490092397 |
| T_CAGATACCAAGCACAG-1 | 3 | Macrophage | Tumor | 0.576283642 |
| T_CAGATTGGTAGCTTTG-1 | 0 | Macrophage | Tumor | 0.387709711 |
| T_CAGATTGGTCAAGCCC-1 | 0 | Macrophage | Tumor | 0.654279236 |
| T_CAGCAATGTGACTGTT-1 | 3 | Macrophage | Tumor | 0.866344001 |
| T_CAGGGCTGTGCATTAC-1 | 1 | Macrophage | Tumor | 0.292782585 |
| T_CAGGTATGTAAGGCCA-1 | 5 | Macrophage | Tumor | 0.368469417 |
| T_CAGTGCGCATTGCCGG-1 | 0 | Macrophage | Tumor | 0.527557536 |
| T_CATAAGCAGAGATTCA-1 | 0 | Macrophage | Tumor | 0.463852706 |
| T_CATAAGCAGCAAGTCG-1 | 0 | Macrophage | Tumor | 0.536182609 |
| T_CATACCCAGCGCCTCA-1 | 0 | Macrophage | Tumor | 0.419136461 |
| T_CATACCCCAATACGCT-1 | 1 | Macrophage | Tumor | 0.405975483 |
| T_CATCCCAAGCGGACAT-1 | 5 | Macrophage | Tumor | 0.514215314 |
| T_CATCGTCAGGTCGTAG-1 | 0 | Macrophage | Tumor | 0.532575443 |
| T_CATCGTCTCACTCGAA-1 | 4 | Macrophage | Tumor | 0.464254163 |
| T_CATTCCGCAGGTCTCG-1 | 0 | Macrophage | Tumor | 0.419018019 |
| T_CATTCTATCAACTGGT-1 | 0 | Macrophage | Tumor | 0.532863499 |
| T_CCACACTTCATCACAG-1 | 0 | Macrophage | Tumor | 0.663660484 |
| T_CCCAACTAGCCATCCG-1 | 0 | Macrophage | Tumor | 0.512015271 |
| T_CCCTGATCACCTAAAC-1 | 1 | Macrophage | Tumor | 0.325785761 |
| T_CCCTTAGGTGGCCCAT-1 | 1 | Macrophage | Tumor | 0.343292203 |
| T_CCGATCTAGTACCCTA-1 | 3 | Macrophage | Tumor | 0.569039488 |
| T_CCTAACCGTCCTCCAT-1 | 1 | Macrophage | Tumor | 0.414549268 |
| T_CCTAAGAAGTAACCTC-1 | 1 | Macrophage | Tumor | 0.36777144 |
| T_CCTAAGAGTAGACTGG-1 | 0 | Macrophage | Tumor | 0.284335049 |
| T_CCTCAACCAATATCCG-1 | 5 | Macrophage | Tumor | 0.349680076 |
| T_CCTCATGTCTTGGAAC-1 | 0 | Macrophage | Tumor | 0.383679683 |
| T_CCTCCTCCACGTACAT-1 | 5 | Macrophage | Tumor | 0.42865228 |
| T_CCTTGTGGTTTGAACC-1 | 0 | Macrophage | Tumor | 0.606611066 |
| T_CGAGAAGCAGGCACAA-1 | 1 | Macrophage | Tumor | 0.589645122 |
| T_CGAGGAAGTTCATCTT-1 | 0 | Macrophage | Tumor | 0.407201389 |
| T_CGCATAAGTACGCTTA-1 | 0 | Macrophage | Tumor | 0.424671507 |
| T_CGCATAATCCTCTAGC-1 | 0 | Macrophage | Tumor | 0.345788448 |
| T_CGCATGGCAAGCACCC-1 | 1 | Macrophage | Tumor | 0.452911181 |
| T_CGCCATTAGTTAACGA-1 | 0 | Macrophage | Tumor | 0.326413172 |
| T_CGGCAGTCATGCCATA-1 | 0 | Macrophage | Tumor | 0.602188373 |
| T_CGGGACTGTAGCGTTT-1 | 1 | Macrophage | Tumor | 0.477042959 |
| T_CGGGACTTCTGGCCGA-1 | 0 | Macrophage | Tumor | 0.632509784 |
| T_CGGGCATCATGTGTCA-1 | 0 | Macrophage | Tumor | 0.4179325 |
| T_CGGGTCACAGATCATC-1 | 3 | Macrophage | Tumor | 0.585359113 |
| T_CGTAATGAGCGCACAA-1 | 0 | Macrophage | Tumor | 0.499284136 |
| T_CGTAGTAAGGCTTTCA-1 | 0 | Macrophage | Tumor | 0.369272902 |
| T_CGTCAAATCTCGCTCA-1 | 0 | Macrophage | Tumor | 0.601654014 |
| T_CGTCCATCAACCCTAA-1 | 1 | Macrophage | Tumor | 0.595354628 |
| T_CGTCCATTCTGCCTCA-1 | 3 | Macrophage | Tumor | 0.38213834 |
| T_CGTGTCTCACCCTAAA-1 | 1 | Macrophage | Tumor | 0.3683749 |
| T_CGTTGGGAGTTGTAGA-1 | 3 | Macrophage | Tumor | 0.563597326 |
| T_CGTTGGGGTTGTGCAT-1 | 0 | Macrophage | Tumor | 0.494465373 |
| T_CGTTGGGGTTTCTATC-1 | 1 | Macrophage | Tumor | 0.539515124 |
| T_CGTTGGGTCAAGAGTA-1 | 0 | Macrophage | Tumor | 0.658140981 |
| T_CTAAGTGTCGACACCG-1 | 0 | Macrophage | Tumor | 0.470740841 |
| T_CTACTATCATGTCAGT-1 | 0 | Macrophage | Tumor | 0.348588702 |
| T_CTATAGGCACCGTGAC-1 | 0 | Macrophage | Tumor | 0.635416235 |
| T_CTATCTAAGGTACCTT-1 | 0 | Macrophage | Tumor | 0.266878799 |
| T_CTCAAGAAGTAGCCAG-1 | 0 | Macrophage | Tumor | 0.386221404 |
| T_CTCATCGGTCATAGTC-1 | 0 | Macrophage | Tumor | 0.687236793 |
| T_CTCCCTCGTCGCCTAG-1 | 5 | Macrophage | Tumor | 0.266290085 |
| T_CTCCGATGTGAATGTA-1 | 0 | Macrophage | Tumor | 0.470996796 |
| T_CTCCTTTGTAGCGCCT-1 | 0 | Macrophage | Tumor | 0.310319653 |
| T_CTGCAGGTCGCCAATA-1 | 5 | Macrophage | Tumor | 0.371251441 |
| T_CTGCCTATCGGCTATA-1 | 0 | Macrophage | Tumor | 0.428257383 |
| T_CTGTCGTAGATCCAAA-1 | 1 | Macrophage | Tumor | 0.639518839 |
| T_CTTACCGGTTGTTGCA-1 | 0 | Macrophage | Tumor | 0.410410861 |
| T_CTTAGGAAGGCCCAAA-1 | 0 | Macrophage | Tumor | 0.42781863 |
| T_CTTCAATTCAGACCTA-1 | 4 | Macrophage | Tumor | 0.262359467 |
| T_CTTCGGTTCTGGGAGA-1 | 1 | Macrophage | Tumor | 0.470812786 |
| T_CTTCTAACAGCATGCC-1 | 5 | Macrophage | Tumor | 0.478983171 |
| T_CTTCTCTTCAACTACG-1 | 3 | Macrophage | Tumor | 0.480067197 |
| T_CTTTCGGTCATTTGTC-1 | 0 | Macrophage | Tumor | 0.390499651 |
| T_GAAATGACAAGCGAGT-1 | 0 | Macrophage | Tumor | 0.389201149 |
| T_GAACTGTTCCAAAGGG-1 | 0 | Macrophage | Tumor | 0.39080121 |
| T_GAAGGGTTCTAGAGCT-1 | 1 | Macrophage | Tumor | 0.316652705 |
| T_GACACGCCACCTCGTT-1 | 0 | Macrophage | Tumor | 0.472650912 |
| T_GACAGCCGTGGCTTAT-1 | 3 | Macrophage | Tumor | 0.595060007 |
| T_GACAGCCGTTAGTTCG-1 | 1 | Macrophage | Tumor | 0.577137101 |
| T_GACCAATAGCGATGAC-1 | 5 | Macrophage | Tumor | 0.36968949 |
| T_GACCTTCGTATAATGG-1 | 0 | Macrophage | Tumor | 0.448071068 |
| T_GACGCTGCAGACAAAT-1 | 0 | Macrophage | Tumor | 0.493821604 |
| T_GACGTTAAGTAAAGCT-1 | 3 | Macrophage | Tumor | 0.646194579 |
| T_GACTCAAAGTAGCCAG-1 | 0 | Macrophage | Tumor | 0.554231463 |
| T_GACTCAAGTACTAACC-1 | 0 | Macrophage | Tumor | 0.565200591 |
| T_GACTCTCGTATGGAAT-1 | 1 | Macrophage | Tumor | 0.434231642 |
| T_GAGAAATCACCTAAAC-1 | 3 | Macrophage | Tumor | 0.420527519 |
| T_GAGCTGCGTGACTAAA-1 | 1 | Macrophage | Tumor | 0.556840333 |
| T_GAGGGATAGCGTGTCC-1 | 5 | Macrophage | Tumor | 0.475135065 |
| T_GAGTGAGCAAGCGCAA-1 | 3 | Macrophage | Tumor | 0.609111413 |
| T_GAGTGAGTCTTCGCTG-1 | 0 | Macrophage | Tumor | 0.449448242 |
| T_GAGTGTTTCTTCGGAA-1 | 0 | Macrophage | Tumor | 0.411033336 |
| T_GAGTTACCATGAATAG-1 | 0 | Macrophage | Tumor | 0.397827788 |
| T_GAGTTGTTCGGCTTGG-1 | 1 | Macrophage | Tumor | 0.35996382 |
| T_GATAGAAGTCTAATCG-1 | 0 | Macrophage | Tumor | 0.392488084 |
| T_GATAGCTCAGATCACT-1 | 1 | Macrophage | Tumor | 0.346553544 |
| T_GATCAGTCACTGCGTG-1 | 1 | Macrophage | Tumor | 0.381589952 |
| T_GATGGAGTCGAGATAA-1 | 1 | Macrophage | Tumor | 0.473252835 |
| T_GATTGGTGTTACGGAG-1 | 0 | Macrophage | Tumor | 0.407239644 |
| T_GATTGGTTCATTATCC-1 | 5 | Macrophage | Tumor | 0.354988127 |
| T_GCAGCTGCAGGTGACA-1 | 0 | Macrophage | Tumor | 0.352058175 |
| T_GCATCGGTCAACCTCC-1 | 0 | Macrophage | Tumor | 0.311513073 |
| T_GCATCTCAGTGAGGTC-1 | 0 | Macrophage | Tumor | 0.376246858 |
| T_GCCCGAAGTTCCTAGA-1 | 0 | Macrophage | Tumor | 0.463201306 |
| T_GCGGATCCACAACGAG-1 | 0 | Macrophage | Tumor | 0.553695475 |
| T_GCGTGCATCTAATTCC-1 | 1 | Macrophage | Tumor | 0.499325858 |
| T_GCTACCTTCTACTGAG-1 | 0 | Macrophage | Tumor | 0.353915131 |
| T_GCTCAAAAGTATAGAC-1 | 1 | Macrophage | Tumor | 0.517075985 |
| T_GCTGGGTCAGACCTGC-1 | 0 | Macrophage | Tumor | 0.349175127 |
| T_GGAATCTCAACAGCCC-1 | 0 | Macrophage | Tumor | 0.513446422 |
| T_GGAGAACGTACAGTCT-1 | 0 | Macrophage | Tumor | 0.490299154 |
| T_GGATCTACATGACGAG-1 | 0 | Macrophage | Tumor | 0.335459879 |
| T_GGATCTATCAGCTCTC-1 | 0 | Macrophage | Tumor | 0.419766784 |
| T_GGATGTTCACGTAGAG-1 | 0 | Macrophage | Tumor | 0.51155094 |
| T_GGCAGTCGTTAGGACG-1 | 4 | Macrophage | Tumor | 0.202761656 |
| T_GGCGTCAAGAGTGTGC-1 | 0 | Macrophage | Tumor | 0.471393134 |
| T_GGCTGTGTCGCATTAG-1 | 3 | Macrophage | Tumor | 0.512996966 |
| T_GGCTTTCGTAATGCGG-1 | 0 | Macrophage | Tumor | 0.348587561 |
| T_GGGACCTAGCGTCTGC-1 | 4 | Macrophage | Tumor | 0.39762413 |
| T_GGGAGATTCTTCTGGC-1 | 1 | Macrophage | Tumor | 0.466754831 |
| T_GGGATGAGTGGTCAAG-1 | 5 | Macrophage | Tumor | 0.418744869 |
| T_GGGCTCAGTTAACCTG-1 | 0 | Macrophage | Tumor | 0.410301897 |
| T_GGGTTTAAGTAGCATA-1 | 0 | Macrophage | Tumor | 0.567673659 |
| T_GGTCTGGAGGTCATTC-1 | 0 | Macrophage | Tumor | 0.447434992 |
| T_GGTGAAGAGCTTAAGA-1 | 5 | Macrophage | Tumor | 0.622554346 |
| T_GGTGAAGAGGTAAGGA-1 | 5 | Macrophage | Tumor | 0.481187744 |
| T_GGTGATTAGGAAGTGA-1 | 0 | Macrophage | Tumor | 0.351725821 |
| T_GGTGGCTTCAAGAGTA-1 | 1 | Macrophage | Tumor | 0.323362794 |
| T_GGTGTTATCCACATAG-1 | 3 | Macrophage | Tumor | 0.627252309 |
| T_GGTGTTATCCAGTGTA-1 | 0 | Macrophage | Tumor | 0.298836761 |
| T_GTAAGTCCAATGAAAC-1 | 1 | Macrophage | Tumor | 0.614715306 |
| T_GTAAGTCGTGGCTGCT-1 | 0 | Macrophage | Tumor | 0.491332704 |
| T_GTAGGTTCACCAAAGG-1 | 0 | Macrophage | Tumor | 0.424600369 |
| T_GTATTTCGTGCGACAA-1 | 1 | Macrophage | Tumor | 0.574685826 |
| T_GTCAAACTCAGATTGC-1 | 0 | Macrophage | Tumor | 0.357893515 |
| T_GTCACTCAGAGGTGCT-1 | 1 | Macrophage | Tumor | 0.639247633 |
| T_GTCACTCTCAATCTTC-1 | 1 | Macrophage | Tumor | 0.332075695 |
| T_GTCATGACAACCCTAA-1 | 1 | Macrophage | Tumor | 0.490006497 |
| T_GTCCACTGTATGAGAT-1 | 1 | Macrophage | Tumor | 0.280712993 |
| T_GTCGAATGTCACGCTG-1 | 5 | Macrophage | Tumor | 0.436258473 |
| T_GTCGAATTCGCTGCGA-1 | 1 | Macrophage | Tumor | 0.529986584 |
| T_GTCGTTCAGGTTCATC-1 | 0 | Macrophage | Tumor | 0.303178678 |
| T_GTCTTTAAGCTCCGAC-1 | 3 | Macrophage | Tumor | 0.771273121 |
| T_GTGAGTTGTTGTAGCT-1 | 1 | Macrophage | Tumor | 0.398237846 |
| T_GTGCAGCGTCGACGCT-1 | 3 | Macrophage | Tumor | 0.48961959 |
| T_GTGCAGCTCACGGGAA-1 | 0 | Macrophage | Tumor | 0.671191131 |
| T_GTGCTGGCATCGGATT-1 | 1 | Macrophage | Tumor | 0.578768535 |
| T_GTGGCGTCAATTCACG-1 | 0 | Macrophage | Tumor | 0.321030438 |
| T_GTGTTAGCAGACTGCC-1 | 0 | Macrophage | Tumor | 0.552596553 |
| T_GTGTTCCTCTGAGATC-1 | 0 | Macrophage | Tumor | 0.522883797 |
| T_GTTACGAAGAGGCTGT-1 | 1 | Macrophage | Tumor | 0.537496639 |
| T_GTTACGACATACCAGT-1 | 1 | Macrophage | Tumor | 0.545789939 |
| T_GTTATGGAGATCGGTG-1 | 1 | Macrophage | Tumor | 0.385181797 |
| T_GTTCCGTAGGTCATCT-1 | 0 | Macrophage | Tumor | 0.371684954 |
| T_GTTCCGTCAGTTGCGC-1 | 0 | Macrophage | Tumor | 0.500722977 |
| T_GTTGAACTCAACCTCC-1 | 3 | Macrophage | Tumor | 0.538504258 |
| T_GTTGTGAGTTACCTTT-1 | 4 | Macrophage | Tumor | 0.425005738 |
| T_GTTTACTCACGCAAAG-1 | 0 | Macrophage | Tumor | 0.352159597 |
| T_TAACCAGGTCTATGAC-1 | 0 | Macrophage | Tumor | 0.518607787 |
| T_TAACTTCAGTGAGGTC-1 | 1 | Macrophage | Tumor | 0.328653852 |
| T_TAACTTCCAGCGTGCT-1 | 0 | Macrophage | Tumor | 0.436498565 |
| T_TAACTTCGTTCCGCAG-1 | 1 | Macrophage | Tumor | 0.425339477 |
| T_TAAGCCACACCCTCTA-1 | 1 | Macrophage | Tumor | 0.255205163 |
| T_TAAGCCATCCGTGTAA-1 | 0 | Macrophage | Tumor | 0.48914705 |
| T_TAAGTCGTCGCTAAAC-1 | 0 | Macrophage | Tumor | 0.498587656 |
| T_TAATTCCCAGGTCTCG-1 | 0 | Macrophage | Tumor | 0.553521654 |
| T_TAATTCCTCGAAACAA-1 | 0 | Macrophage | Tumor | 0.438152939 |
| T_TACCCGTAGTTCTACG-1 | 1 | Macrophage | Tumor | 0.428357645 |
| T_TACCGAAAGTCATGAA-1 | 0 | Macrophage | Tumor | 0.427176763 |
| T_TAGCACAGTATCGCGC-1 | 0 | Macrophage | Tumor | 0.430605323 |
| T_TAGCACAGTGGATCAG-1 | 1 | Macrophage | Tumor | 0.389629076 |
| T_TAGGAGGCAGCGGATA-1 | 3 | Macrophage | Tumor | 0.657157649 |
| T_TAGTGCATCCACACCT-1 | 3 | Macrophage | Tumor | 0.409953593 |
| T_TATACCTTCCTATTTG-1 | 0 | Macrophage | Tumor | 0.428013543 |
| T_TATACCTTCGTAGAGG-1 | 0 | Macrophage | Tumor | 0.436165588 |
| T_TATATCCTCTCAATCT-1 | 3 | Macrophage | Tumor | 0.726100987 |
| T_TATCAGGGTTTCGGCG-1 | 0 | Macrophage | Tumor | 0.378146397 |
| T_TATCCTACAGGTTCAT-1 | 5 | Macrophage | Tumor | 0.48761639 |
| T_TATGTTCAGTTACGTC-1 | 0 | Macrophage | Tumor | 0.267300229 |
| T_TATTCCAAGAATAACC-1 | 1 | Macrophage | Tumor | 0.406711414 |
| T_TATTCCACATACAGGG-1 | 5 | Macrophage | Tumor | 0.315456772 |
| T_TCAATTCCAGAGAGGG-1 | 1 | Macrophage | Tumor | 0.545984341 |
| T_TCAGCCTCATATTCGG-1 | 1 | Macrophage | Tumor | 0.508349895 |
| T_TCAGGGCAGACGCCAA-1 | 0 | Macrophage | Tumor | 0.401737296 |
| T_TCAGTCCAGTTGGGAC-1 | 5 | Macrophage | Tumor | 0.328579833 |
| T_TCAGTGAAGGACGCAT-1 | 1 | Macrophage | Tumor | 0.481003949 |
| T_TCAGTTTTCACGGACC-1 | 5 | Macrophage | Tumor | 0.344076824 |
| T_TCATACTCACGTCATA-1 | 1 | Macrophage | Tumor | 0.274419583 |
| T_TCATATCAGTCCTGCG-1 | 3 | Macrophage | Tumor | 0.408825243 |
| T_TCATATCCAACACTAC-1 | 3 | Macrophage | Tumor | 0.36535774 |
| T_TCATATCCAATTGCTG-1 | 1 | Macrophage | Tumor | 0.521273817 |
| T_TCATTGTGTTGCTCAA-1 | 0 | Macrophage | Tumor | 0.449235039 |
| T_TCCACCAGTGACACAG-1 | 1 | Macrophage | Tumor | 0.388922658 |
| T_TCCATCGCAGGCACTC-1 | 0 | Macrophage | Tumor | 0.553123143 |
| T_TCCATCGTCTATTTCG-1 | 1 | Macrophage | Tumor | 0.471314028 |
| T_TCCCATGGTCAACACT-1 | 1 | Macrophage | Tumor | 0.486104836 |
| T_TCCGATCTCCTCAGAA-1 | 0 | Macrophage | Tumor | 0.482790148 |
| T_TCCGTGTTCTTGGTGA-1 | 1 | Macrophage | Tumor | 0.474809576 |
| T_TCCTCGAAGATGCTTC-1 | 0 | Macrophage | Tumor | 0.475051136 |
| T_TCCTCGAAGGTCGTCC-1 | 0 | Macrophage | Tumor | 0.437897959 |
| T_TCCTCGATCGGTCGAC-1 | 0 | Macrophage | Tumor | 0.359367932 |
| T_TCGACCTTCTTGCAGA-1 | 1 | Macrophage | Tumor | 0.465002583 |
| T_TCGACGGAGTAGGAAG-1 | 0 | Macrophage | Tumor | 0.471998122 |
| T_TCGGATATCCTAGAGT-1 | 1 | Macrophage | Tumor | 0.543135423 |
| T_TCGGGCAGTACGATCT-1 | 4 | Macrophage | Tumor | 0.400878996 |
| T_TCTACCGAGGGCAACT-1 | 3 | Macrophage | Tumor | 0.507845741 |
| T_TCTACCGTCTCATTGT-1 | 3 | Macrophage | Tumor | 0.526671084 |
| T_TCTATACCAGAAGCTG-1 | 0 | Macrophage | Tumor | 0.487239628 |
| T_TCTATCAAGCTGACCC-1 | 1 | Macrophage | Tumor | 0.201995875 |
| T_TCTCACGAGCGTATGG-1 | 1 | Macrophage | Tumor | 0.596581054 |
| T_TCTCACGGTCGGTAAG-1 | 5 | Macrophage | Tumor | 0.422354096 |
| T_TCTCCGATCCACGTGG-1 | 1 | Macrophage | Tumor | 0.454684812 |
| T_TCTGTCGGTGACTGTT-1 | 0 | Macrophage | Tumor | 0.3823724 |
| T_TCTTCCTTCGGTAGGA-1 | 0 | Macrophage | Tumor | 0.532277915 |
| T_TCTTGCGAGAGTCTTC-1 | 0 | Macrophage | Tumor | 0.521286133 |
| T_TGACGCGAGAGAACCC-1 | 0 | Macrophage | Tumor | 0.532752282 |
| T_TGAGCATAGGCGTTAG-1 | 0 | Macrophage | Tumor | 0.442715315 |
| T_TGAGGTTCATGAGGGT-1 | 0 | Macrophage | Tumor | 0.563513933 |
| T_TGAGTCATCTGTTGGA-1 | 0 | Macrophage | Tumor | 0.391660943 |
| T_TGATGCATCCTAACAG-1 | 0 | Macrophage | Tumor | 0.50889841 |
| T_TGATGGTAGTCCCAAT-1 | 4 | Macrophage | Tumor | 0.298550458 |
| T_TGCAGATGTTATGTCG-1 | 0 | Macrophage | Tumor | 0.50265544 |
| T_TGCGACGCAAGTACCT-1 | 3 | Macrophage | Tumor | 0.683557977 |
| T_TGCGGGTCACCTGATA-1 | 0 | Macrophage | Tumor | 0.325499805 |
| T_TGCTCGTTCGCGATCG-1 | 5 | Macrophage | Tumor | 0.420152733 |
| T_TGGAGAGAGACATAAC-1 | 1 | Macrophage | Tumor | 0.440533429 |
| T_TGGATGTAGAATCGAT-1 | 0 | Macrophage | Tumor | 0.492817214 |
| T_TGGATGTAGGATAATC-1 | 5 | Macrophage | Tumor | 0.373960617 |
| T_TGGGCTGCAGGCACAA-1 | 1 | Macrophage | Tumor | 0.354544473 |
| T_TGGGTTAGTTTGGAGG-1 | 3 | Macrophage | Tumor | 0.564680553 |
| T_TGTAACGCATGACAGG-1 | 0 | Macrophage | Tumor | 0.520198869 |
| T_TGTACAGGTGTGCCTG-1 | 0 | Macrophage | Tumor | 0.365723635 |
| T_TGTAGACCATCGAACT-1 | 1 | Macrophage | Tumor | 0.576110516 |
| T_TGTTGGACAATGAAAC-1 | 1 | Macrophage | Tumor | 0.497803351 |
| T_TGTTGGATCTGCACCT-1 | 3 | Macrophage | Tumor | 0.634192629 |
| T_TTAATCCAGAGGTTTA-1 | 5 | Macrophage | Tumor | 0.305403152 |
| T_TTAATCCGTCCCAAAT-1 | 0 | Macrophage | Tumor | 0.357586659 |
| T_TTACGTTAGAAGCGAA-1 | 0 | Macrophage | Tumor | 0.568270409 |
| T_TTAGGGTTCTGCGGCA-1 | 0 | Macrophage | Tumor | 0.437163501 |
| T_TTAGTCTAGGAGTCTG-1 | 0 | Macrophage | Tumor | 0.440030769 |
| T_TTCACCGGTAACAGGC-1 | 1 | Macrophage | Tumor | 0.676826431 |
| T_TTCAGGAGTGAACGGT-1 | 0 | Macrophage | Tumor | 0.45173438 |
| T_TTCATGTGTTAAAGTG-1 | 1 | Macrophage | Tumor | 0.627166617 |
| T_TTCCTAATCTTACGTT-1 | 0 | Macrophage | Tumor | 0.379308585 |
| T_TTCTTCCAGAATGTTG-1 | 1 | Macrophage | Tumor | 0.510761942 |
| T_TTGAGTGAGTTCGCAT-1 | 0 | Macrophage | Tumor | 0.446873222 |
| T_TTGCCTGCACATTCGA-1 | 0 | Macrophage | Tumor | 0.430016744 |
| T_TTGGGCGAGGTCTACT-1 | 1 | Macrophage | Tumor | 0.745528536 |
| T_TTGGGTAGTGAATTGA-1 | 0 | Macrophage | Tumor | 0.374195042 |
| T_TTGTGGACAGCAGTGA-1 | 0 | Macrophage | Tumor | 0.446344495 |
| T_TTGTGGAGTTCAAAGA-1 | 1 | Macrophage | Tumor | 0.372107922 |
| T_TTGTGTTTCTAGTTCT-1 | 0 | Macrophage | Tumor | 0.516516367 |
| T_TTGTTCAGTATCCTTT-1 | 0 | Macrophage | Tumor | 0.4654176 |
| T_TTGTTTGCACCCTTGT-1 | 5 | Macrophage | Tumor | 0.207717915 |
| T_TTTATGCAGGAGCAAA-1 | 0 | Macrophage | Tumor | 0.398731582 |
